# Interrupted-Sampling Repeater Jamming Suppression for Frequency-Agile Radar Based on Time–Frequency Analysis and Multi-Domain Filtering

**DOI:** 10.3390/s26216752

**Published:** 2026-10-25

**Authors:** Fan Xu, Feng Luo, Linrang Zhang, Zixuan He, Jiahao Han, Wanyao Wu, Lin Wang

**Affiliations:** 1 National Laboratory of Radar Signal Processing, Xidian University, Xi’an 710071, China; 21021110339@stu.xidian.edu.cn (F.X.); luofeng@xidian.edu.cn (F.L.); lrzhang@xidian.edu.cn (L.Z.); hanjiahao@xidian.edu.cn (J.H.); 24021110045@stu.xidian.edu.cn (W.W.); linwang111@stu.xidian.edu.cn (L.W.); 2 The 54th Research Institute of China Electronics Technology Group Corporation, Shijiazhuang 050081, China

**Keywords:** frequency-agile radar, interrupted-sampling repeater jamming, time–frequency analysis, radar jamming suppression, multi-domain filtering

## Abstract

**Highlights:**

**What are the main findings?**

- For joint intra-pulse and inter-pulse frequency-agile waveforms, interrupted-sampling repeater jamming (ISRJ) components exhibit joint time–frequency offsets relative to genuine target echoes because each forwarded segment retains the frequency of the previously sampled sub-pulse.
- The proposed time- and frequency-domain decision criteria distinguish jamming-contaminated cells from target-dominated cells, enabling two-dimensional time–frequency filtering to suppress different ISRJ modes while limiting losses in target energy and pulse-compression gain.

**What are the implications of the main findings?**

- Sub-pulse-wise time–frequency filtering based on probability density reduces inadvertent removal of target components, preserving target energy for subsequent radar processing.
- The improved suppression performance across broad signal-to-noise ratio (SNR) and jamming-to-signal ratio (JSR) ranges indicates that the method has the potential to preserve target-bearing echoes in radar remote-sensing systems operating under low-SNR and strong-jamming conditions.

**Abstract:**

Interrupted-sampling repeater jamming (ISRJ) produces coherent false targets after pulse compression and degrades the reliability of frequency-agile radar sensors, particularly at low signal-to-noise ratio (SNR) and high jamming-to-signal ratio (JSR). Existing single-domain suppression methods often provide insufficient separation between jamming and target echoes and may attenuate target-bearing components. This paper proposes a joint time–frequency analysis and multi-domain filtering method for radar waveforms employing intra-pulse and inter-pulse frequency agility. A forwarded jamming segment retains the carrier frequency of the sampled sub-pulse while overlapping a subsequent sub-pulse with a different carrier frequency, producing a joint displacement in time and frequency relative to the genuine target echo. Based on this property, a sub-pulse-wise two-dimensional feature extraction strategy is developed in the short-time Fourier transform (STFT) domain. Empirical probability density criteria are derived from projections along the time and frequency axes to determine adaptive rejection thresholds. The resulting time-selective and frequency-selective rejection regions are combined through logical intersection to construct a two-dimensional notch filter. The retained components are reconstructed using the inverse STFT and processed by segmented pulse compression. Simulations covering direct, periodic, and cyclic ISRJ show that the proposed method suppresses coherent false targets while limiting the loss of pulse-compression gain. It achieves higher peak signal-to-jamming-plus-noise ratios than the reference method over the tested SNR and JSR ranges and remains effective under adverse conditions. The proposed method improves the interference resilience of frequency-agile radar sensors and provides more reliable target echoes for subsequent detection and parameter estimation.

## 1. Introduction

Interrupted-sampling repeater jamming (ISRJ) is a coherent deception-jamming technique implemented using digital radio frequency memory (DRFM). Its principal modes include interrupted-sampling direct repeater jamming (ISDRJ), interrupted-sampling periodic repeater jamming (ISPRJ), and interrupted-sampling cyclic repeater jamming (ISCRJ). In these modes, the transmitted radar waveform is sampled intermittently and retransmitted to generate multiple coherent false targets around the genuine target. ISRJ combines low sampling-rate and storage requirements with a rapid response and strong deception capability. It can cause severe target–jamming overlap or dense false-target distributions, degrading radar detection and parameter-estimation performance [1,2,3,4]. In radar remote-sensing systems used for target detection in electromagnetically contested environments, these false targets can obscure weak returns and reduce the reliability of subsequent detection and parameter estimation. Effective ISRJ suppression must therefore preserve useful target components and coherent processing gain.

ISRJ suppression methods can be broadly classified into waveform design and transmitter optimization, signal reconstruction and cancellation, blind source separation, transform-domain processing, and intelligent computing approaches. Waveform design and transmitter optimization methods employ intra-pulse stepped-frequency waveforms or joint intra- and inter-pulse frequency-agile waveforms to improve the separation between genuine target echoes and jamming signals and enhance anti-jamming capability at the transmitter [5,6,7,8,9,10,11]. Signal reconstruction and cancellation methods estimate or reconstruct the jamming components and subtract them from the received echoes to recover the genuine target signal [12,13,14,15,16,17,18]. Blind source separation methods exploit differences between the distributions of target echoes and jamming signals in feature space to separate the signals and detect targets [19,20,21]. Transform-domain processing methods use differences between target and jamming signals in the time, frequency, or other transform domains to suppress interference through filtering or component extraction [22,23,24,25,26]. Jamming recognition and suppression methods based on intelligent computing and deep learning have also attracted increasing attention in recent years.

Existing ISRJ suppression methods have three main limitations. First, poor separation between target and jamming echoes in a single domain may cause conventional filters to attenuate or remove target components, making the target undetectable. Second, frequency-agile radar commonly uses threshold segmentation or binarization to separate genuine echoes from dense false targets after exploiting inter-pulse frequency agility. Conventional criteria such as Otsu [27,28,29] and Kittler–Illingworth [30] adapt poorly to changes in the echo-amplitude distribution with JSR. An unsuitable threshold therefore discards genuine echoes or retains excessive jamming, particularly at high JSR. Third, these methods lack robustness at low SNR or high JSR, with marked performance degradation when both conditions occur.

To address these limitations, this paper considers radar signals employing joint intra-pulse and inter-pulse frequency-agile modulation. The temporal discontinuity and periodic forwarding of ISRJ, together with mutual masking between sub-pulses caused by random or pseudorandom intra-pulse frequency hopping, create a joint time–frequency displacement between the jamming and genuine target echoes. The proposed method therefore extracts two-dimensional features for each sub-pulse in the short-time Fourier transform (STFT) domain. It combines time- and frequency-domain criteria to construct a two-dimensional filter that selectively removes jamming-contaminated cells. This design preserves target-bearing components and limits the loss of pulse-compression gain after suppression.

The remainder of this paper is organized as follows. Section 2 establishes the frequency-agile radar echo and ISRJ models and outlines the limitations of the existing suppression methods used for comparison. Section 3 presents the proposed time–frequency analysis and multi-domain filtering method. Section 4 presents simulation results and performance analysis. Section 5 discusses the implications and limitations of the results. Section 6 concludes the paper.

## 2. Signal Model and Existing Suppression Methods

### 2.1. Frequency-Agile Radar Echo and ISRJ Model

This study considers a radar waveform with joint inter-pulse and intra-pulse frequency agility, which provides the time–frequency separability required by the proposed method. A coherent processing interval (CPI) contains *M* pulses. Each pulse comprises *N_S_* linearly frequency-modulated (LFM) sub-pulses with a width of *T_s_* and a bandwidth of *B_s_*. The duration and bandwidth of each pulse are *T_p_* = *N_S_T_s_* and *B_p_* = *N_S_B_s_*, respectively. The *n_s_*-th sub-pulse of the *m*-th pulse can be expressed as

(1) $$\begin{array}{c} s\left(t,m,n_{s}\right)=\mathrm{rect}\left(\frac{t-\left(n_{s}-1\right)T_{s}}{T_{s}}\right)\exp\left(j2\pi b(n_{s})\Delta f_{s}t\right)\\ \cdot \exp\left(j\pi \gamma {\left(t-\left(n_{s}-1\right)T_{s}\right)}^{2}\right) \end{array}$$

where the window function is

(2) $$\mathrm{rect}\left(\frac{t}{T_{s}}\right)=\left\{\begin{array}{cc} 1, & |t|⩽\frac{T_{s}}{2}\\ 0, & \mathrm{else} \end{array}\right.$$

where *γ* = *B_s_*/*T_s_* is the chirp rate, Δ*f_s_* is the sub-pulse frequency step, and *b*(*n_s_*) is the known sub-pulse frequency code, with *b*(*n_s_*) ∈ {0,1,…,*U*} and *U* ≥ *N_S_*. The indices are *m* = 1,…,*M* and *n_s_* = 1,…,*N_S_*. The carrier frequency of pulse *m* is *f_m_* = *f*_0_ + *a*(*m*)Δ*f*, where *f*_0_ is the reference carrier, *a*(*m*) is the known pulse frequency code, and Δ*f* is the inter-pulse frequency step. Define the transmitted complex envelope as $q_{m}\left(t\right)=\sum_{n_{s}=1}^{N_{S}}s\left(t,m,n_{s}\right)$. The transmitted radio-frequency analytic signal of pulse *m* is

(3) $$S_{T}^{m}\left(t\right)=q_{m}\left(t\right)e^{j2\pi f_{m}\left[t+\left(m-1\right)T_{r}\right]}$$

where *T_r_* is the pulse repetition period. The fast-time coordinate *t* is measured from the center of the first sub-pulse, consistent with (1) and (2); *t* + (*m* − 1)*T_r_* is the corresponding time in the pulse train. The same coordinate and carrier reference are used for transmission, reception, and downconversion.

Assume that there are *G* moving point targets within the observation range. The initial range and radial velocity of the *g*-th target are denoted by *r_g_* and *v_g_*, respectively, with *g* = 1,…,*G*. Positive radial velocity denotes motion toward the radar. Under the stop-and-go approximation, the range is held constant within each pulse and updated between pulses as $R_{g}^{m}=r_{g}-v_{g}\left(m-1\right)T_{r}$. The corresponding round-trip delay is $\tau_{g}^{m}=2R_{g}^{m}/c$, where *c* is the speed of light. The target echo of the *m*-th pulse can be expressed as

(4) $$S_{R,\mathrm{RF}}^{m}\left(t\right)=\sum_{g=1}^{G}\sigma_{g}S_{T}^{m}\left(t-\tau_{g}^{m}\right)$$

where *σ_g_* is the complex scattering coefficient of target *g*. The subscript RF distinguishes the radio-frequency analytic echo from its complex baseband envelope. Mixing with the conjugate carrier reference $e^{-j2\pi f_{m}\left[t+\left(m-1\right)T_{r}\right]}$ gives

(5) $$\begin{array}{c} S_{R}^{m}\left(t\right)=S_{R,\mathrm{RF}}^{m}\left(t\right)e^{-j2\pi f_{m}\left[t+\left(m-1\right)T_{r}\right]}\\ =\sum_{g=1}^{G}\sigma_{g}e^{-j2\pi f_{m}\tau_{g}^{m}}q_{m}\left(t-\tau_{g}^{m}\right). \end{array}$$

A single DRFM-based ISRJ source is considered. Because no spatial processing is performed, jammer angular coordinates are not required. Let $\tau_{J}^{m}$ denote the total radar-to-jammer and jammer-to-radar propagation delay. A sampled fragment is indexed by l, and its retransmission by *k*. The window $w_{l}^{m}\left(u\right)=\mathrm{rect}\left[\left(u-u_{l}^{m}\right)/T_{\mathrm{sam}}\right]$ selects a fragment of duration *T*_sam_, centered at $u_{l}^{m}$ on the transmitted-envelope time axis. The additional storage and forwarding delay is $d_{lk}^{m}$, giving the total delay $D_{lk}^{m}=\tau_{J}^{m}+d_{lk}^{m}$. The complex gain $\eta_{lk}^{m}$ includes propagation loss, replay gain, and any fixed replay phase. The set $A^{m}$ lists the fragment–retransmission pairs in the forwarding schedule. In the same baseband domain as (5), the mixed received signal is

(6) $$\begin{array}{l} S_{\mathrm{RJ}}^{m}\left(t\right)=S_{R}^{m}\left(t\right)+J^{m}\left(t\right)+n^{m}\left(t\right),\\ J^{m}\left(t\right)=\sum_{\left(l,k\right)\in A^{m}}\eta_{lk}^{m}e^{-j2\pi f_{m}D_{lk}^{m}}w_{l}^{m}\left(t-D_{lk}^{m}\right)q_{m}\left(t-D_{lk}^{m}\right). \end{array}$$

where *n^m^*(*t*) is complex Gaussian white receiver noise. Each sample window and the sampled waveform are delayed together, and each propagation or forwarding delay is included once through $D_{lk}^{m}$. The target delay $\tau_{g}^{m}$ is not reused as a jammer delay. For periodic interrupted sampling, the window centers are separated by a sampling period *T_J_*; the sampling duty factor is *T*_sam_/*T_J_*. This repetition rate 1/*T_J_* is distinct from the radar digitization rate, and *T*_sam_ is distinct from the radar sub-pulse width *T_s_*. The forwarding delays must satisfy the causal timing of the selected schedule.

Equation (6) represents the three ISRJ modes through $A^{m}$ and $d_{lk}^{m}$. ISDRJ includes one retransmission for each sampled fragment. ISPRJ includes *Q* retransmissions of the same fragment; successive copies are separated by the specified replay interval, so $d_{l,k+1}^{m}-d_{lk}^{m}$ equals that interval. After each sampled fragment, ISCRJ replays the most recently stored fragments, at most *C* of them, in their original order. Thus, the mode is specified by both the selected pairs and their replay times. The repeated-forwarding count in Section 4 is *Q* = 3, and the cyclic-forwarding count is *C* = 2 (Appendix A lists all jammer settings); *G* is reserved for the number of targets. For reference, define the baseband matched-filter kernel $h_{m}\left(t\right)=q_{m}^{*}\left(-t\right)$. Its pulse-compression output is

(7) $$\begin{array}{ll} S_{\mathrm{pc}}^{m}\left(t\right) & =\left(S_{\mathrm{RJ}}^{m}⊗h_{m}\right)\left(t\right)\\ & =\left(S_{R}^{m}⊗h_{m}\right)\left(t\right)+\left(J^{m}⊗h_{m}\right)\left(t\right)\\ & +\left(n^{m}⊗h_{m}\right)\left(t\right). \end{array}$$

where ⊗ denotes convolution. Pulse compression acts on the complete received waveform; a sampling window cannot in general be moved outside this convolution. Equation (7) defines the corresponding unfiltered matched-filter output. The suppression procedure in Section 3 instead applies time–frequency filtering to the complex baseband mixture in (6), followed by the segmented matched filtering in (23) and (24).

**Figure 1:**
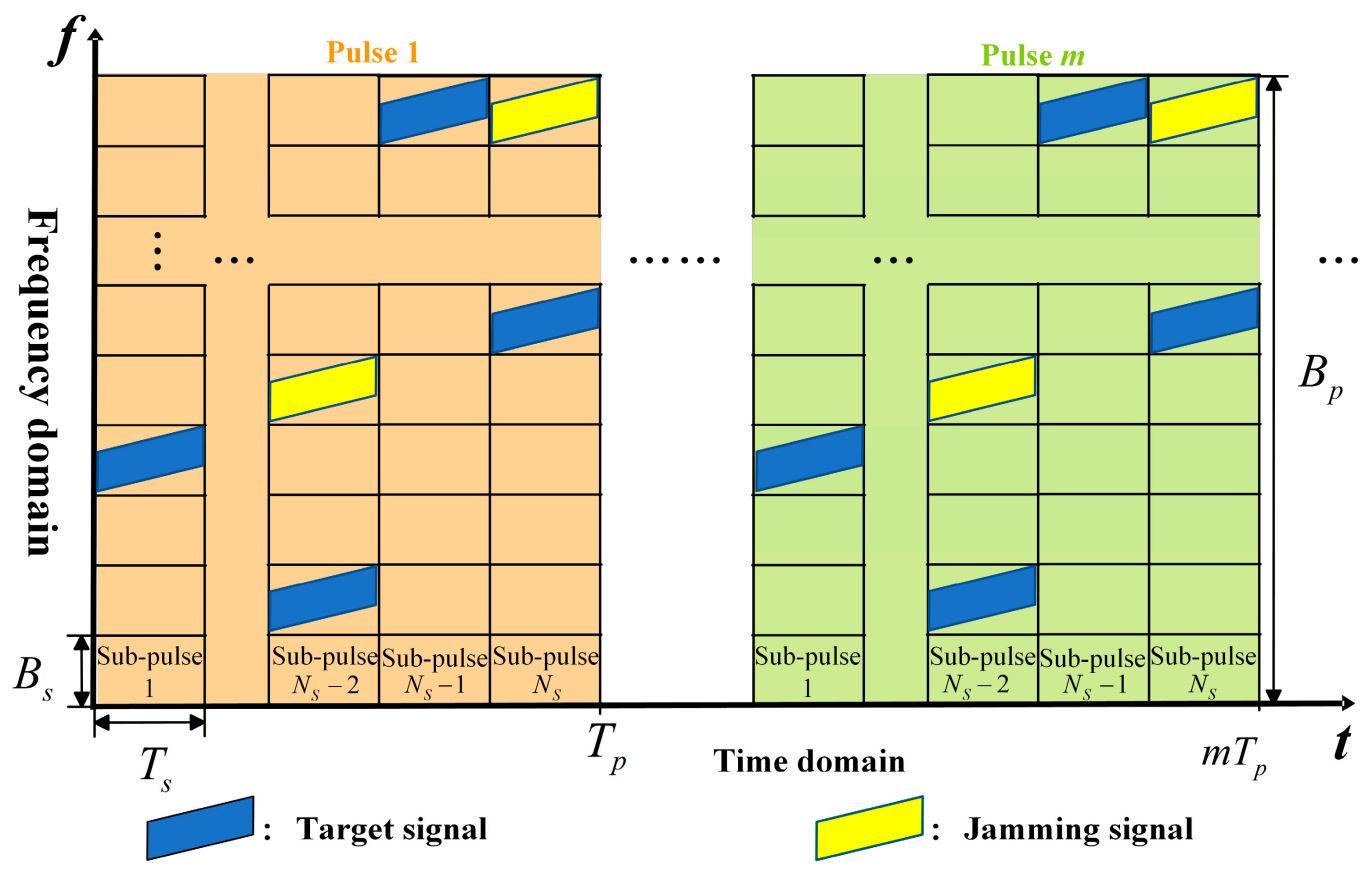
Illustration of received signals.

To illustrate the time–frequency relationship between the target echo and ISRJ, the received signal is partitioned into sub-pulses of duration *T_s_*, as shown in Figure 1.

### 2.2. Limitations of Existing Suppression Methods

Three classes of existing methods are directly relevant to the waveform in Section 2.1 and serve as references in the remainder of this paper; their processing steps and limitations are outlined below. The first is time–frequency filtering with a one-dimensional mask [9]. The STFT is applied to each complete received pulse, and its magnitude is summed over frequency to obtain a time projection. A single threshold is selected from the projection values of the CPI using the Otsu criterion [27,28,29], which maximizes the between-class variance of the two classes separated by the threshold. STFT frames whose projection exceeds the threshold are removed over the entire frequency axis, and the remaining components are reconstructed by the inverse STFT and processed by segmented pulse compression. Because a frame is removed as a whole, target components that share the frame with a forwarded fragment are removed together with the jamming, and because the threshold follows the class statistics, it shifts with the JSR. Related criteria, such as Kittler–Illingworth minimum-error thresholding [30], can replace the Otsu criterion in the same structure.

The second class reconstructs and cancels the jamming. Sparse-reconstruction methods such as [31] estimate the sampling and forwarding parameters of the jammer over a dictionary, reconstruct the jamming, and subtract it from the received signal, so their performance depends on the accuracy of the estimated parameters. The third class is learning-based: the method in [32] trains a recurrent network to locate the jamming-free segments of the echo and builds a filter from them, which requires training data that represent the waveform and the jamming conditions.

To address these limitations, this paper suppresses ISRJ by two-dimensional time–frequency filtering of each sub-pulse. The thresholds on the time and frequency projections are derived from the modal intervals of their empirical amplitude histograms rather than from a two-class criterion, and a time–frequency cell is rejected only when both directional decisions flag it, so that a flagged frame is not removed over the entire frequency axis. Neither estimates of the jammer parameters nor training data are required. The numerical comparison with [9] and with alternative threshold criteria, together with the computational cost, is given in Section 4.4, which also revisits the limitations of the second and third classes.

## 3. Algorithm

**Figure 2:**
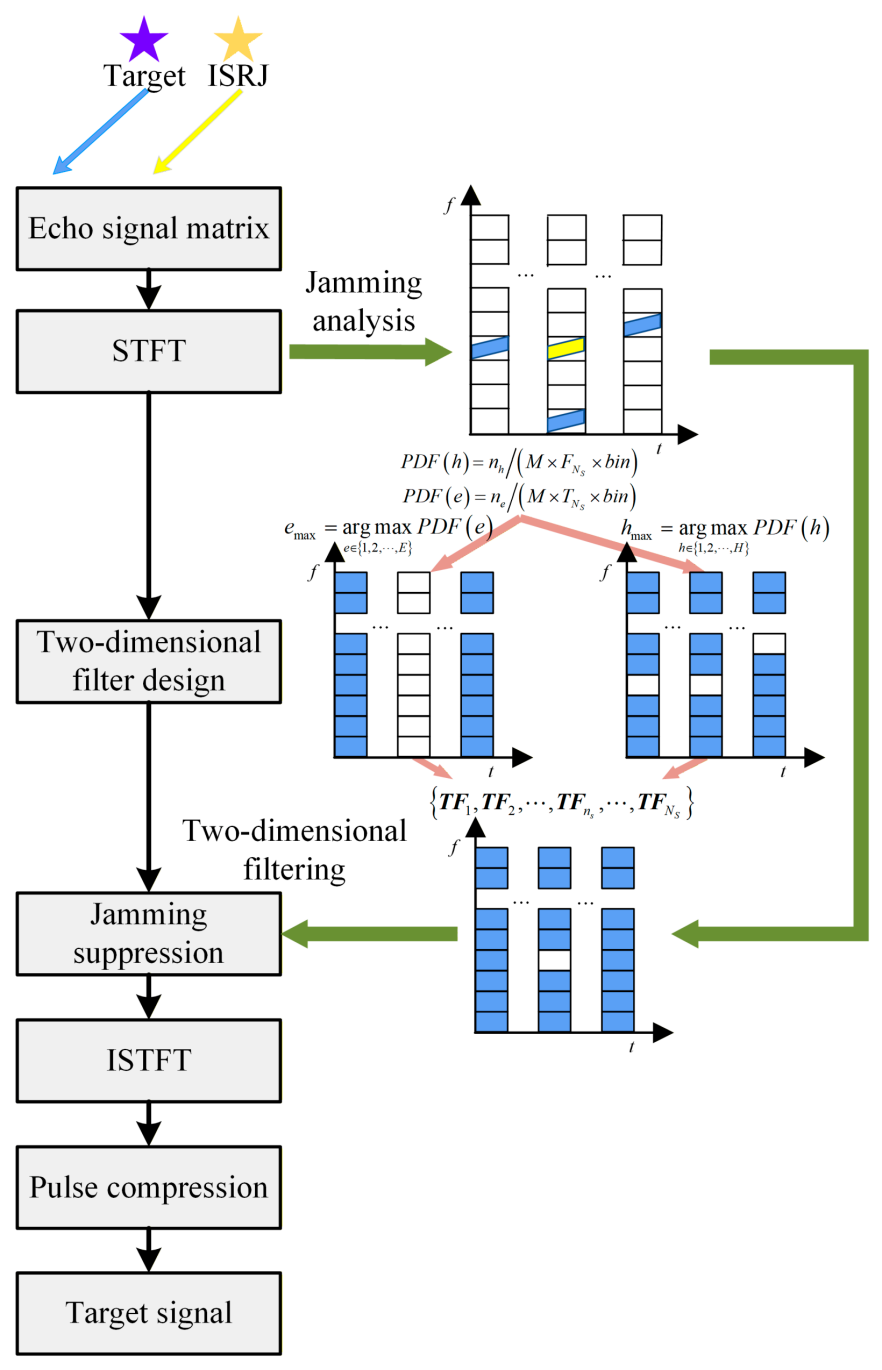
Flowchart of the proposed ISRJ suppression method.

This section develops the proposed ISRJ suppression method based on time–frequency analysis and multi-domain filtering. The processing flow is shown in Figure 2; the inverse short-time Fourier transform (ISTFT) and probability density function (PDF) used in the procedure are defined below.

### 3.1. Time–Frequency Analysis Based on the Short-Time Fourier Transform

For radar signals with joint inter-pulse and intra-pulse frequency agility, a forwarded ISRJ segment retains the carrier frequency of an earlier sampled sub-pulse while overlapping a later sub-pulse with a different carrier frequency. This mechanism produces the joint time–frequency displacement described in Section 2. The STFT [33,34,35] provides a linear complex time–frequency representation of the received echo. Its component STFTs add linearly, as in (9), although taking the magnitude still introduces dependence on their relative phases.

According to (6), write the mixed baseband echo as $s^{m}\left(t\right)=S_{\mathrm{RJ}}^{m}\left(t\right)$ for the STFT processing below:

(8) $$s^{m}\left(t\right)=s_{r}^{m}\left(t\right)+s_{j}^{m}\left(t\right)+s_{n}^{m}\left(t\right)$$

where $s_{r}^{m}\left(t\right)=S_{R}^{m}\left(t\right)$, $s_{j}^{m}\left(t\right)=J^{m}\left(t\right)$, and $s_{n}^{m}\left(t\right)=n^{m}\left(t\right)$ denote the target, jamming, and noise components, respectively. Their STFTs satisfy

(9) $$\begin{array}{ll} {\mathrm{STFT}}_{s}^{m}\left(t,f\right) & =\int_{-\infty }^{\infty }s^{m}\left(u\right)\omega \left(u-t\right)e^{-j2\mathrm{\pi fu}}\mathrm{du}\\ & ={\mathrm{STFT}}_{r}^{m}\left(t,f\right)+{\mathrm{STFT}}_{j}^{m}\left(t,f\right)+{\mathrm{STFT}}_{n}^{m}\left(t,f\right) \end{array}$$

where *u* is the integration variable, *ω* is the real analysis window, and the three terms on the second line are the complex STFTs of the target, jamming, and noise components. The variables *t* and *f* in (9) are the continuous analysis time and frequency; the discrete STFT uses a symmetric 16-sample Hamming window *ω*, an 8-sample hop, and an *N_F_* = 2048-point FFT, giving *K* = 499 frames per pulse repetition interval.

The STFT of the jamming signal exhibits a sinc-shaped distribution in the frequency domain, with considerable sidelobe energy. When threshold segmentation is used to suppress interference in the time–frequency domain, a trade-off therefore arises: a threshold that is too low causes target loss, whereas a threshold that is too high retains the jamming sidelobes and leaves residual interference. Moreover, removing the sub-pulse segments affected by jamming also removes the target signal contained in them, which reduces the integration gain of segmented pulse compression.

### 3.2. ISRJ Suppression Based on Two-Dimensional Time–Frequency Filtering

To suppress ISRJ while preserving the target echo, a two-dimensional (2-D) time–frequency notch filter is designed for each sub-pulse. Separate time- and frequency-domain masks identify cells contaminated by jamming, and their joint use limits interference between adjacent sub-pulse regions.

#### 3.2.1. Time-Domain Filter Construction

The mixed baseband echo in (8) is divided into the *N_S_* sub-pulse components by time gates referenced to a common alignment instant *t*_0_:

(10) $$s^{m,n_{s}}\left(t\right)=s^{m}\left(t\right)\mathrm{rect}\left(\frac{t-t_{0}-\left(n_{s}-1\right)T_{s}}{T_{s}}\right),n_{s}=1,\ldots ,N_{S}$$

where *t*_0_ is the center of the first echo sub-pulse and rect(⋅) is defined in (2). Applying the STFT in (9) gives ${\mathrm{STFT}}^{m,n_{s}}\left(t,f\right)$.

The time-domain projection vector $t_{s}^{m,n_{s}}\left(t\right)$ for the *n_s_*-th sub-pulse is obtained by summing the STFT magnitude matrix $\left|{\mathrm{STFT}}^{m,n_{s}}\left(t,f\right)\right|$ over frequency:

(11) $$t_{s}^{m,n_{s}}\left(t\right)=\sum_{f=1}^{F_{N_{S}}}\left|{\mathrm{STFT}}^{m,n_{s}}\left(t,f\right)\right|$$

Here, $t=1,\ldots ,T_{N_{S}}$ and $f=1,\ldots ,F_{N_{S}}$ index the *K* time frames and the *N_F_* frequency bins of the discrete STFT matrix (Section 3.1), respectively. The projection is computed from the magnitude of the mixed received-signal STFT. The complex STFT components add linearly as in (9), but their magnitudes do not generally add; no component-wise magnitude decomposition is used in (11). Observable time–frequency contrast between target-bearing and jamming-contaminated regions motivates the projection features, but the definition in (11) does not require component-wise magnitude additivity.

The time-domain projection vectors for the *n_s_*-th sub-pulse, $t_{s}^{m,n_{s}}\left(t\right)$, are arranged across the *M* pulses to form a matrix $T_{n_{s}}$ of size $T_{N_{S}}\times M$. Each entry $T_{n_{s}}\left(t,m\right)$ gives the projection magnitude at the *t*-th sampling instant for the *n_s_*-th sub-pulse of the *m*-th pulse.

Collect the maximum projection amplitudes of all sub-pulse matrices into *T*_max_, expressed as

(12) $$T_{\max}=\left\{T_{\max}^{1},T_{\max}^{2},\cdots ,T_{\max}^{n_{s}},\cdots ,T_{\max}^{N_{S}}\right\}$$

where $T_{\max}^{n_{s}}$ is the largest projection amplitude in $T_{n_{s}}$, with $t=1,\ldots ,T_{N_{S}}$ and *m* = 1,…,*M*:

(13) $$T_{\max}^{n_{s}}=\max_{t,m}\left\{T_{n_{s}}\left(t,m\right)\right\}$$

Using (12) and (13), the minimum value of *T*_max_ is defined as the unit sub-interval width *win_T_* for the sub-interval division, given by

*win_T_* = min{*T*_max_} (14)

Using the amplitude range and width scale defined in (13) and (14), the matrix $T_{n_{s}}$ is divided into *E* sub-intervals within interval $\left[0,T_{\max}^{n_{s}}\right]$, where $E=\max\left\{1,\left\lfloor T_{\max}^{n_{s}}/\mathrm{wins}+1/2\right\rfloor \right\}$, *wins* = *α_T_win_T_* is the nominal sub-interval width, and *α_T_* is the width multiplier of Section 4.2.1. The actual sub-interval width is $\mathrm{bin}=T_{\max}^{n_{s}}/E$. With a fixed nominal width, *E* is adaptively calculated under various JSR conditions, and the left and right boundaries of each sub-interval are expressed as

(15) $$\left\{\begin{array}{l} L_{e}=\left(e-1\right)\times \mathrm{bin}\\ R_{e}=e\times \mathrm{bin} \end{array}\right.,e=1,2,\cdots ,E$$

where *L_e_* and *R_e_* are the left and right boundaries, respectively, of the *e*-th sub-interval.

Each histogram interval includes its left boundary and excludes its right boundary, except the final interval, which includes both boundaries. In (16), *I_e_* denotes interval *e*, and $1_{I_{e}}$ is its indicator function. Thus, every matrix entry, including repeated amplitudes and samples on shared boundaries, is counted exactly once.

Using the interval boundaries in (15), the number of elements in $T_{n_{s}}$ that fall into the *e*-th sub-interval is given by

(16) $$n_{e}=\overset{T_{N_{S}}}{\underset{t=1}{\sum }}\overset{M}{\underset{m=1}{\sum }}1_{I_{e}}\left(T_{n_{s}}\left(t,m\right)\right)$$

where *n_e_* counts the entries of $T_{n_{s}}$ that lie in interval *I_e_*. The upper boundary *R_e_* represents that interval when choosing the threshold. The empirical histogram density (PDF) is

(17) $$\mathrm{PDF}\left(e\right)=\frac{n_{e}}{\left(M\times T_{N_{S}}\times \mathrm{bin}\right)}$$

For equal positive bin widths, multiplying PDF(*e*) by the actual bin width gives the probability mass of interval e; summing these masses over all intervals gives one.

According to (17), the upper boundary of the sub-interval with the largest PDF(*e*) is used as the segmentation threshold; in case of a tie, the lowest one is used. This threshold is denoted by $t_{n_{s}}^{*}$ for $T_{n_{s}}$. Repeating the above procedure for the matrices $T_{n_{s}}$ corresponding to all sub-pulses yields the threshold set $\left\{t_{1}^{*},t_{2}^{*},\cdots ,t_{n_{s}}^{*},\cdots ,t_{N_{S}}^{*}\right\}$. The common time-domain segmentation threshold *t*^∗^ is then given by

(18) $$t^{*}=\min\left\{t_{1}^{*},t_{2}^{*},\cdots ,t_{n_{s}}^{*},\cdots ,t_{N_{S}}^{*}\right\}$$

With the threshold *t*^∗^, the matrix $T_{n_{s}}$ can be binarized as follows:

(19) $$T_{n_{s}}^{\prime }\left(t,m\right)=\left\{\begin{array}{ll} 1, & \left|T_{n_{s}}\left(t,m\right)\right|\leq t^{*}\\ 0, & \left|T_{n_{s}}\left(t,m\right)\right|>t^{*} \end{array}\right.$$

Processing the *N_S_* sub-pulses yields the matrix set $\left\{{Τ}_{1}^{\prime },{Τ}_{2}^{\prime },\cdots ,{Τ}_{n_{s}}^{\prime },\cdots ,{Τ}_{N_{S}}^{\prime }\right\}$. These binary matrices act as time-domain band-stop filters for the corresponding sub-pulse echoes in the time–frequency domain.

#### 3.2.2. Frequency-Domain Filter Construction

The frequency-domain filter is constructed in the same way as the time-domain filter, with the projection taken onto the frequency axis to form the matrix $F_{n_{s}}$ of size $F_{N_{S}}\times M$. The unit sub-interval width of this projection, obtained as in (12)–(14), is denoted by *win_F_*. The segmentation threshold set $\left\{f_{1}^{*},f_{2}^{*},\cdots ,f_{n_{s}}^{*},\cdots ,f_{N_{S}}^{*}\right\}$ is obtained using the same PDF-based criterion, with the nominal width *α_F_ win_F_*. To account for the spread of chirp-signal energy across frequency, the common frequency-domain threshold *f*^∗^ is given by

(20) $$f^{*}=\sum_{i=1}^{N_{S}}\frac{f_{i}^{*}}{N_{S}}$$

Subsequently, the binary matrix $F_{n_{s}}^{\prime }$ is given by

(21) $$F_{n_{s}}^{\prime }\left(f,m\right)=\left\{\begin{array}{ll} 1, & \left|F_{n_{s}}\left(f,m\right)\right|\leq f^{*}\\ 0, & \left|F_{n_{s}}\left(f,m\right)\right|>f^{*} \end{array}\right.$$

where $F_{n_{s}}(f,m)$ is the magnitude in the *f*-th frequency bin of the frequency-domain projection for the *n_s_*-th sub-pulse of the *m*-th pulse. The matrix set $\left\{F_{1}^{\prime },F_{2}^{\prime },\cdots ,F_{n_{s}}^{\prime },\cdots ,F_{N_{S}}^{\prime }\right\}$ is obtained by processing the *N_S_* sub-pulses. Similarly, the resultant binarized matrix corresponds to a frequency-domain band-stop filter for each sub-pulse echo in the time–frequency domain.

The modal histogram interval provides a data-dependent estimate of the dominant low-amplitude background, and its upper boundary identifies potentially contaminated projection samples. The minimum time threshold keeps the time-direction criterion active as the jamming level varies, while the mean frequency threshold accounts for the spread of the LFM spectrum. The two rejection regions are intersected, so a time–frequency cell is rejected only when both criteria identify it as contaminated. Thus, widening the time-direction region alone does not remove target-bearing cells. This conservative rule limits inadvertent target removal, although residual jamming may remain.

#### 3.2.3. Two-Dimensional Time–Frequency Filter Construction

Based on the time-domain binary matrix set $\left\{{Τ}_{1}^{\prime },{Τ}_{2}^{\prime },\cdots ,{Τ}_{n_{s}}^{\prime },\cdots ,{Τ}_{N_{S}}^{\prime }\right\}$ and the frequency-domain binary matrix set $\left\{F_{1}^{\prime },F_{2}^{\prime },\cdots ,F_{n_{s}}^{\prime },\cdots ,F_{N_{S}}^{\prime }\right\}$, a two-dimensional time–frequency filter is constructed.

For sub-pulse *n_s_* of pulse *m*, define the binary time–frequency mask $TF_{n_{s}}^{m}\in {\left\{0,1\right\}}^{T_{N_{S}}\times F_{N_{S}}}$ by

(22) $$TF_{n_{s}}^{m}\left(t,f\right)=1-\left[1-T_{n_{s}}^{\prime }\left(t,m\right)\right]\left[1-F_{n_{s}}^{\prime }\left(f,m\right)\right]$$

where $T_{n_{s}}^{\prime }\left(t,m\right)$ and $F_{n_{s}}^{\prime }\left(f,m\right)$ are the time- and frequency-direction decisions for the same pulse, with 1 indicating retention and 0 indicating rejection. The indices are *m* = 1,…,*M*, $t=1,\ldots ,T_{N_{S}}$, and $f=1,\ldots ,F_{N_{S}}$. Thus, (22) rejects a cell only when both directional decisions are zero; it takes the intersection of their rejection regions, or equivalently the logical OR of their retention decisions.

The time-direction decision identifies instants with elevated projected magnitude, while the frequency-direction decision identifies occupied frequency bins. The joint time–frequency displacement of forwarded fragments motivates combining this evidence. Equation (22) rejects only cells flagged by both criteria; this is an operational decision rule, not an assertion of perfect separation between target and jamming. Rejecting the union would also remove cells flagged in only one direction. The intersection retains more coefficients, limiting inadvertent target-energy removal at the cost of possible residual jamming.

Repeat the above procedure to obtain $\left\{TF_{1}^{m},\ldots ,TF_{N_{S}}^{m}\right\}$ for each pulse *m*. Element-wise multiplication of each mask with the corresponding original complex STFT matrix retains the phase of the selected coefficients and yields an estimate of the target-bearing time–frequency matrix. The mask family therefore differs from pulse to pulse, which is why the pulse index appears in (22).

Finally, the inverse short-time Fourier transform (ISTFT) reconstructs the retained time–frequency components in the time domain, yielding an estimate of the target-bearing echo $s^{\prime m,n_{s}}\left(t\right)$. The ISTFT uses the same window, hop, and FFT length as the STFT.

### 3.3. Segmented Pulse Compression

Because the LFM sub-pulses have different frequency offsets, matched filtering is applied separately to each known sub-pulse waveform rather than to the sub-pulses as a single LFM segment. This segmented pulse-compression procedure uses one matched filter per sub-pulse [36,37,38]. The aligned sub-pulse outputs are summed to obtain the final pulse-compression result. The matched-filter output for each sub-pulse is expressed as

(23) $$y\left(t,m,n_{s}\right)=\left(s^{\prime m,n_{s}}⊗h_{m,n_{s}}\right)\left(t\right)$$

where $s^{\prime m,n_{s}}\left(t\right)$ is the reconstructed complex echo from Section 3.2.3, and $h_{m,n_{s}}\left(t\right)=s^{*}\left(-t,m,n_{s}\right)$ is the matched-filter kernel for the known transmitted sub-pulse in (1). The superscript * denotes complex conjugation, and ⊗ denotes convolution. Equivalently, the output is the correlation $y\left(t,m,n_{s}\right)=\int_{-\infty }^{\infty }s^{\prime m,n_{s}}\left(u\right)s^{*}\left(u-t,m,n_{s}\right)\mathrm{du}$. The received segment and its reference use the same fast-time origin, so their correlation outputs share a common delay coordinate *t* before summation. Any local segment indexing must be converted to this common coordinate. The pulse-compression output for pulse *m* is

(24) $$y\left(t,m\right)=\sum_{n_{s}=1}^{N_{S}}y\left(t,m,n_{s}\right)$$

For the single-target case, write *R* = *r*_1_, *v* = *v*_1_, and $\tau^{m}=\tau_{1}^{m}=2\left[R-v\left(m-1\right)T_{r}\right]/c$, following the delay convention in Section 2. The propagation phase in (5) therefore satisfies the identity below. Its velocity-dependent term has a positive sign for a target approaching the radar. Equation (24) retains the full delay-dependent output; a time-independent peak-phase expression cannot represent *y*(*t*,*m*) over all fast-time samples.

(25) $$e^{-j2\pi f_{m}\tau^{m}}=e^{-j4\pi f_{m}R/c}e^{+j4\pi f_{m}v\left(m-1\right)T_{r}/c}$$

To interpret the aligned peak, consider an ideal single-target echo with correctly matched transmitted sub-pulse references and a common delay coordinate, excluding residual jamming, receiver noise, and filtering distortion. Under these assumptions, the known sub-pulse frequency code appears in both the echo and reference in (23) and cancels at the correlation peak. The sub-pulse contributions then share the propagation phase and combine coherently:

(26) $$y\left(\tau^{m},m\right)=e^{-j4\pi f_{m}R/c}e^{+j4\pi f_{m}v\left(m-1\right)T_{r}/c}\sum_{n_{s}=1}^{N_{S}}A_{m,n_{s}}$$

where $A_{m,n_{s}}$ denotes the sub-pulse peak coefficient after factoring out the displayed propagation phase. Under the ideal single-target assumptions, these coefficients share the target’s scattering phase and add coherently within each pulse. The leading phase depends on the carrier of pulse *m* and hence on the inter-pulse frequency code, which explains the inter-pulse phase incoherence discussed in Section 4. The ideal peak relation does not describe arbitrary noisy or distorted filtered outputs; those retain the full dependence given in (24).

The output of (24) is a range–pulse matrix, and the range profile assessed in Section 4 is obtained from it by one further step, stated here so that the quantity appearing in (28) is unambiguous. Writing *y*(*τ*,*m*) for the output of (24) and *ρ_m_* for the complex sample at its largest-magnitude range peak in pulse *m*, the matrix is first reduced across the coherent processing interval to a single range response by the peak-weighted average:

(27) $$w\left(\tau \right)=\frac{\sum_{m}y\left(\tau ,m\right)\rho_{m}^{*}}{\sum_{m}{\left|\rho_{m}\right|}^{2}}$$

This response is multiplied by the pulse-compressed received signal obtained by applying (23) and (24) without suppression. The resulting product is the range profile evaluated in Section 4 and used in (28). Thus, the unfiltered pulse-compressed profile is weighted to emphasize range cells where the suppressed signal retains energy.

## 4. Simulation Experiments

This section validates the proposed ISRJ suppression method through simulations based on the model in Section 2. The radar and target parameters are listed in Table 1, and the jammer settings in Table A1 (Appendix A). Throughout this section, the SNR is the ratio of the target-echo power to the receiver-noise power at the receiver input, evaluated over the pulse duration before pulse compression, with the noise power taken over the 100 MHz sampling bandwidth. Segmented pulse compression raises the SNR by 29.0 dB for the parameters in Table 1; an input SNR between −22 and −2 dB therefore corresponds to 7–27 dB after pulse compression. The JSR is the ratio of the jamming power to the target-echo power at the receiver input over the pulse duration. Unless otherwise specified, the SNR is −7.0 dB and the JSR is 17.0 dB for ISDRJ. Because the three forwarding schedules occupy different fractions of the pulse, the same jammer setting gives a JSR of 18.8 dB for ISPRJ and 17.0 dB for ISCRJ (16.4 dB in Section 4.1). Where the JSR is swept, the abscissa is the jamming-to-signal power ratio within the forwarded segments, which exceeds the pulse-averaged JSR by 3.0, 1.2, and 3.0 dB for ISDRJ, ISPRJ, and ISCRJ, respectively.

The parameters in Table 1 are representative of a typical X-band frequency-agile pulse radar. The pulse structure (64 pulses of 8 μs, each formed by eight LFM sub-pulses of 1 μs and 6 MHz), the inter-pulse frequency step, and the pulse repetition frequency are the same as in the reference method [9], and the remaining values were selected to meet the conditions that the processing requires. The sub-pulse frequency step of 5 MHz and the sub-pulse frequency code set the frequency separation between a forwarded fragment and the sub-pulse that it overlaps, on which the method relies, and the sampling frequency of 100 MHz covers the occupied band. The inter-pulse frequency step of 9 MHz with a codebook of 128 entries gives an agile span of about 1.15 GHz, and the pulse repetition frequency of 25 kHz gives an unambiguous range of 6 km, which contains the simulated target. The same parameters are used for the reference method and for all threshold variants. Other waveform parameter sets were not simulated; the operating conditions on which the performance mainly depends (the SNR, the JSR, the ISRJ mode, and the jammer replay latency) and the sub-interval widths are varied in Section 4.1, Section 4.2, Section 4.3 and Section 4.4.

### 4.1. Suppression Results

**Figure 3:**
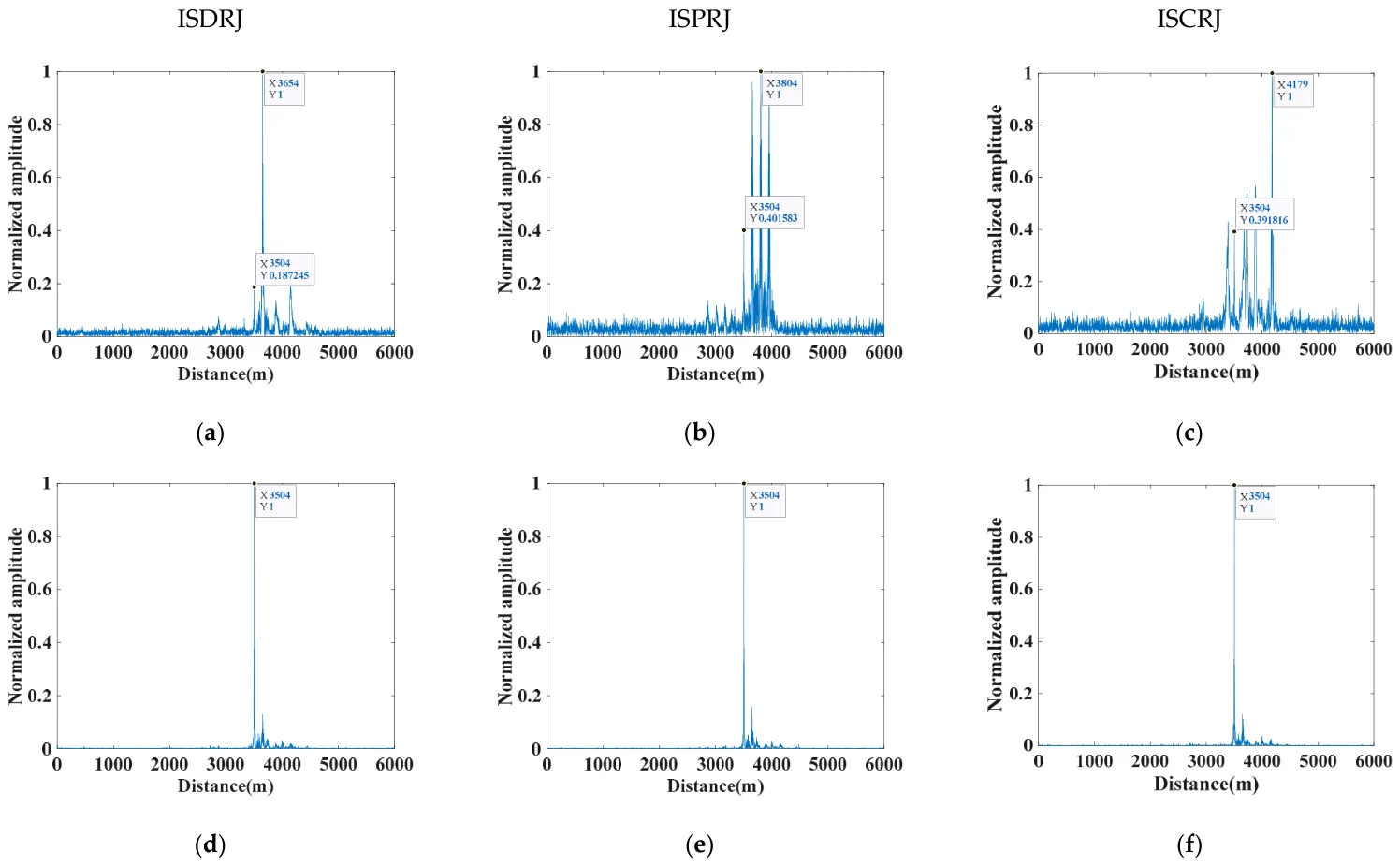
Pulse-compression results of a single pulse for the three ISRJ modes. (**a**–**c**) Without multi-domain filtering: ISDRJ, ISPRJ, and ISCRJ. (**d**–**f**) With multi-domain filtering: ISDRJ, ISPRJ, and ISCRJ.

**Figure 4:**
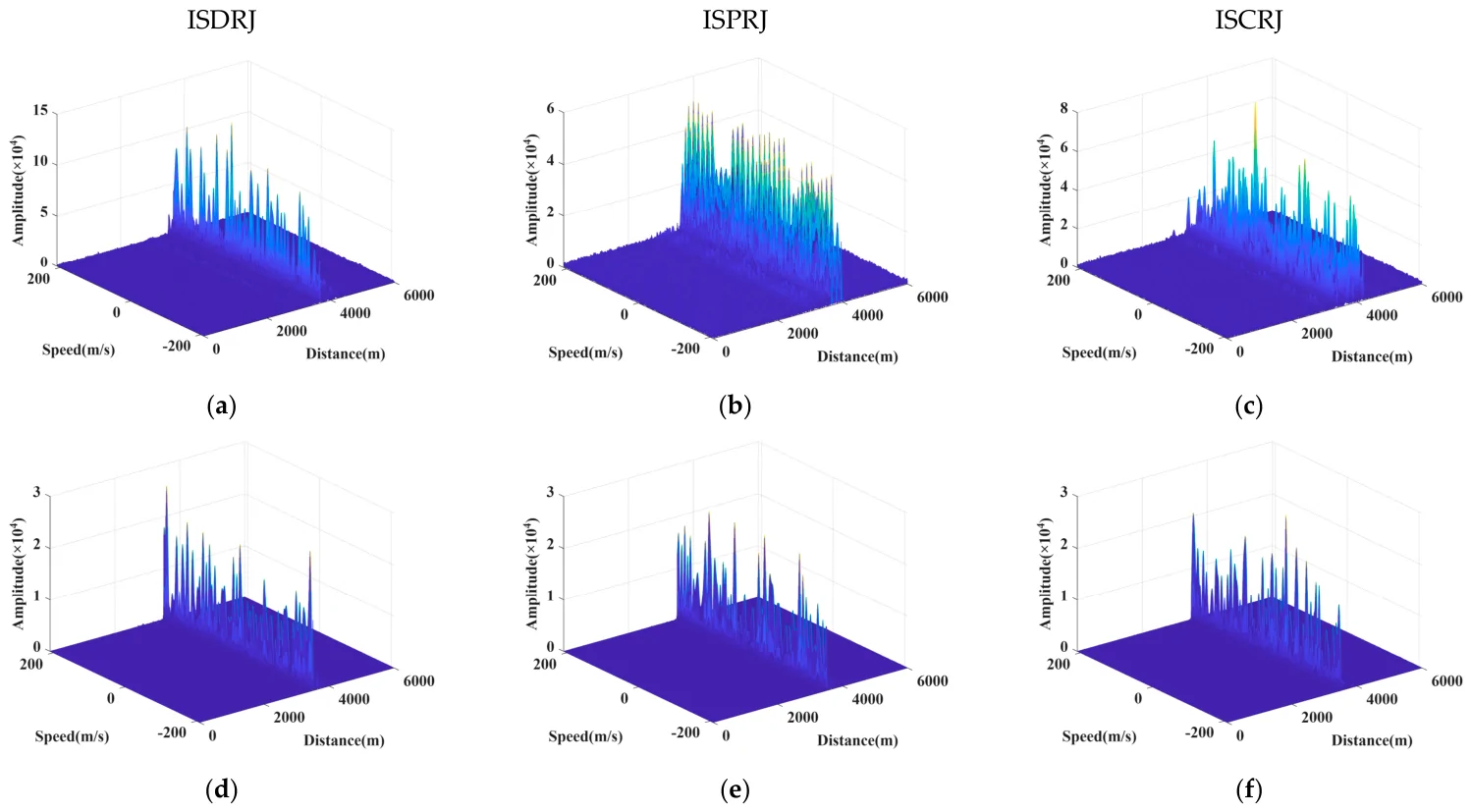
Coherent processing results after full-pulse compression for the three ISRJ modes, shown as three-dimensional surfaces. (**a**–**c**) Without multi-domain filtering: ISDRJ, ISPRJ, and ISCRJ. (**d**–**f**) With multi-domain filtering: ISDRJ, ISPRJ, and ISCRJ.

**Figure 5:**
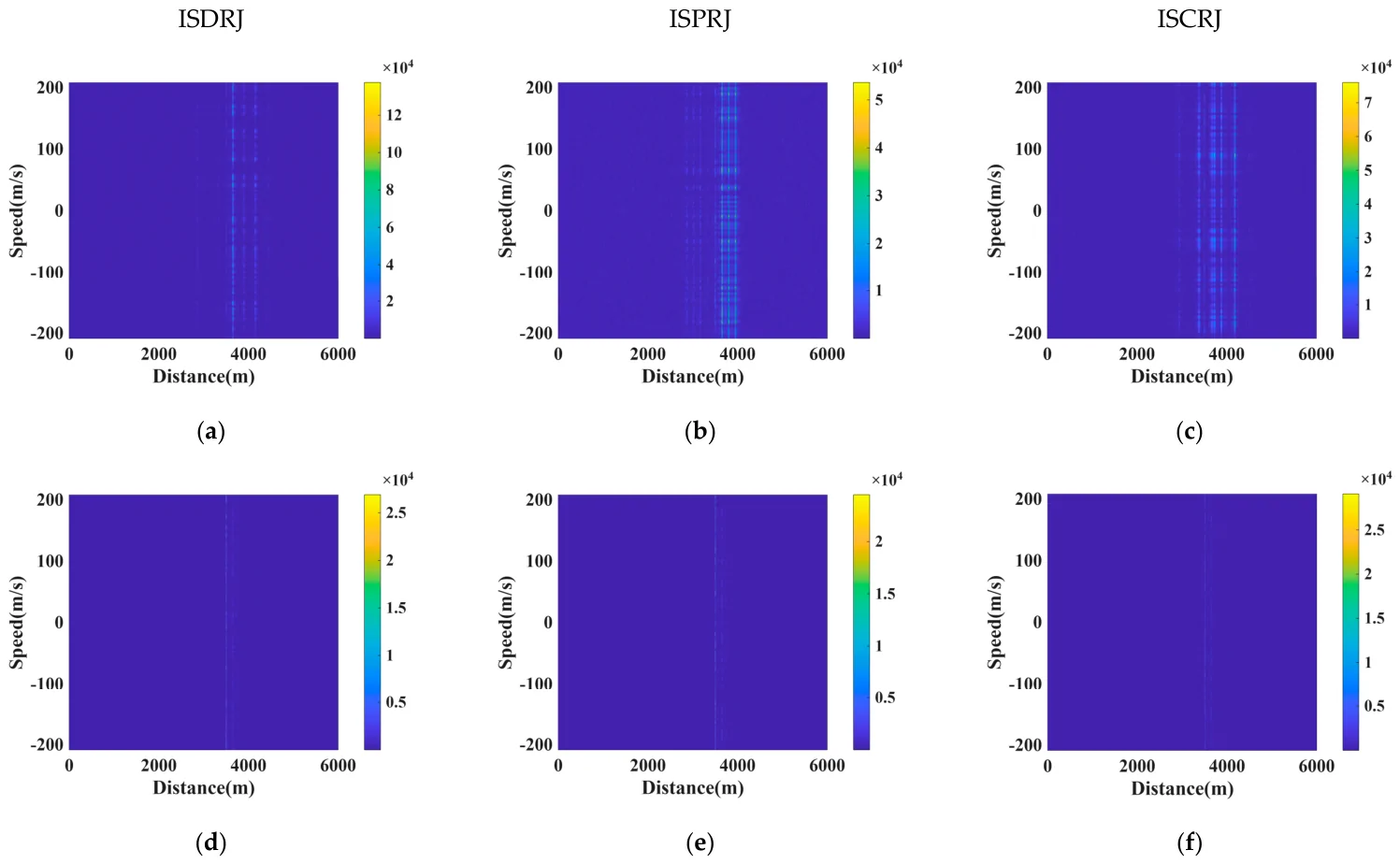
Coherent processing results after full-pulse compression for the three ISRJ modes, shown as distance–speed maps. (**a**–**c**) Without multi-domain filtering: ISDRJ, ISPRJ, and ISCRJ. (**d**–**f**) With multi-domain filtering: ISDRJ, ISPRJ, and ISCRJ.

**Figure 6:**
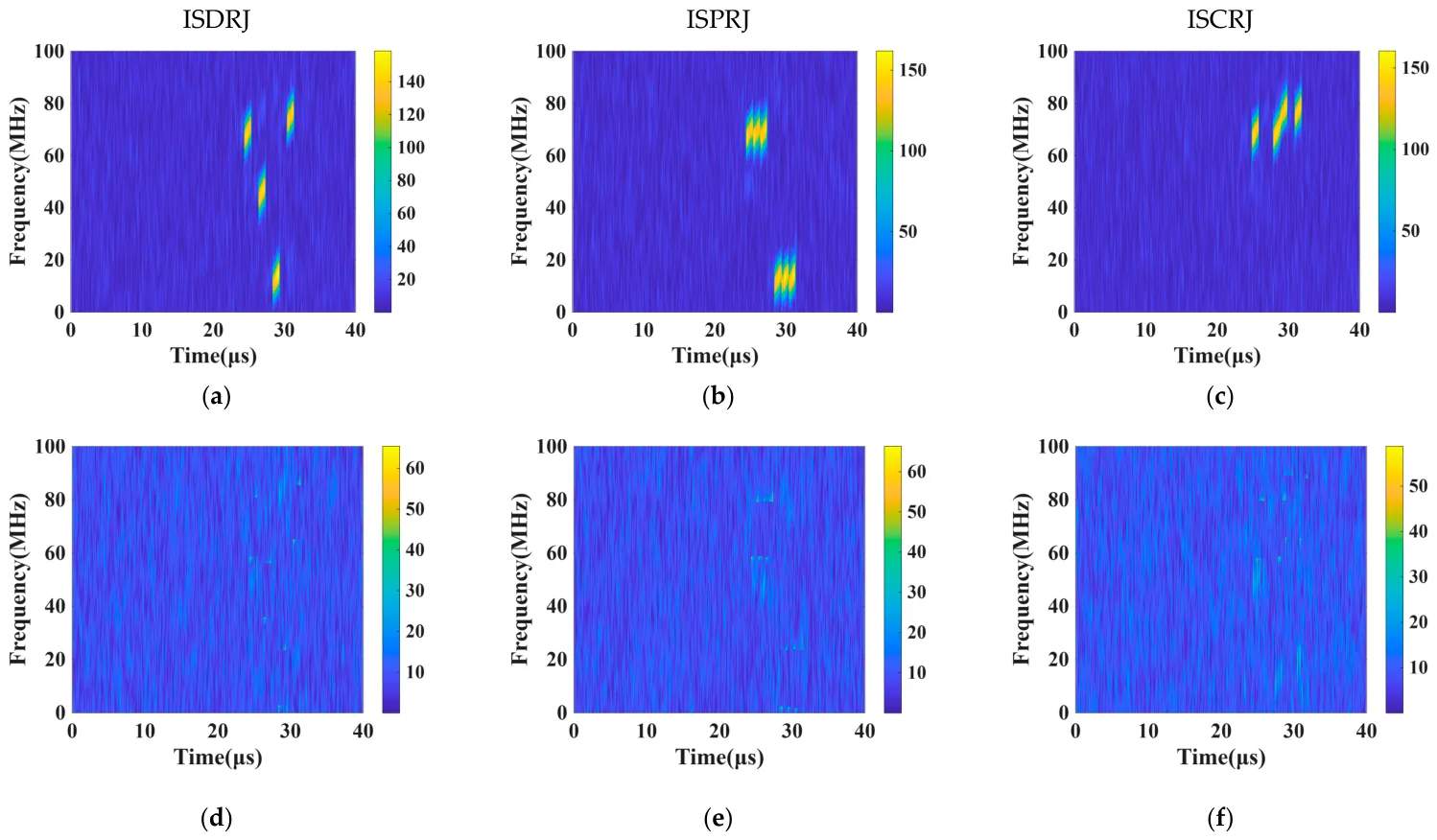
Time–frequency distributions of the received signal with receiver noise for the three ISRJ modes. (**a**–**c**) Before suppression: ISDRJ, ISPRJ, and ISCRJ. (**d**–**f**) After suppression: ISDRJ, ISPRJ, and ISCRJ.

**Figure 7:**
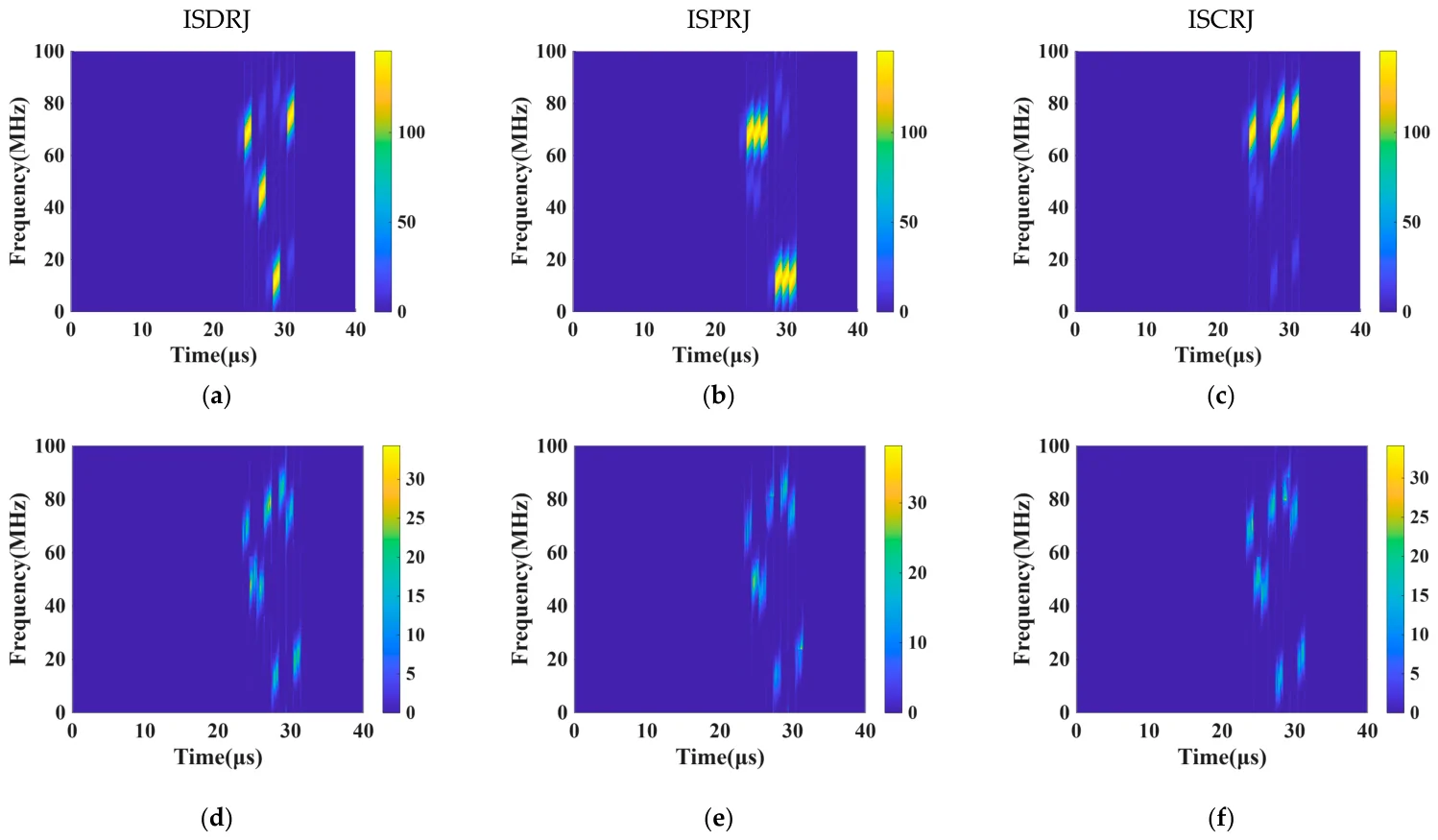
Time–frequency distributions of the received signal without receiver noise for the three ISRJ modes. (**a**–**c**) Before suppression: ISDRJ, ISPRJ, and ISCRJ. (**d**–**f**) After suppression: ISDRJ, ISPRJ, and ISCRJ.

Figure 3, Figure 4, Figure 5, Figure 6 and Figure 7 present the suppression results for the three ISRJ modes side by side. In each figure, the columns correspond to ISDRJ, ISPRJ with a repeated-forwarding count of three, and ISCRJ with a cyclic-forwarding count of two, and the two rows show the results before and after the proposed multi-domain filtering. All results use the parameters in Table 1.

Figure 3 shows the pulse-compression result of a single pulse. Without filtering, the strongest peak in each mode belongs to a false target, at 3654 m for ISDRJ, 3804 m for ISPRJ, and 4179 m for ISCRJ, whereas the true target at 3504 m reaches only 0.19, 0.40, and 0.39 of that peak, respectively. After filtering, the strongest peak is at the true target range in all three modes, and the residual false-target peaks do not exceed 0.16 of the target peak.

Figure 4 and Figure 5 show the results after coherent processing of the 64 pulses, as three-dimensional surfaces and as distance–speed maps. Before filtering, the false targets form several ridges around the target, between about 3400 and 4200 m. After filtering, the response is concentrated at the target distance in all three modes. Because the inter-pulse frequency agility makes the phase incoherent from pulse to pulse, direct coherent processing does not focus the target along the speed axis, with or without filtering, so the target parameters cannot be read from these maps.

Figure 6 and Figure 7 show the time–frequency distributions of the received signal with and without receiver noise. Before suppression, the forwarded fragments appear as strong components that are displaced in time and frequency from the target sub-pulses. With receiver noise (Figure 6), the jamming components are removed by the filter, and the remaining target components are difficult to distinguish from the noise background. Without receiver noise (Figure 7), the jamming components are removed while the target components are preserved.

### 4.2. Performance Analysis

This section analyzes the key factors affecting the performance of the proposed jamming suppression method and compares it with existing approaches. The analysis focuses on: (1) the influence of sub-interval width on segmented pulse-compression gain; (2) the effect of jammer replay latency; (3) jamming suppression performance under different JSRs; and (4) performance under different SNRs.

#### 4.2.1. Sub-Interval Width Analysis

**Figure 8:**
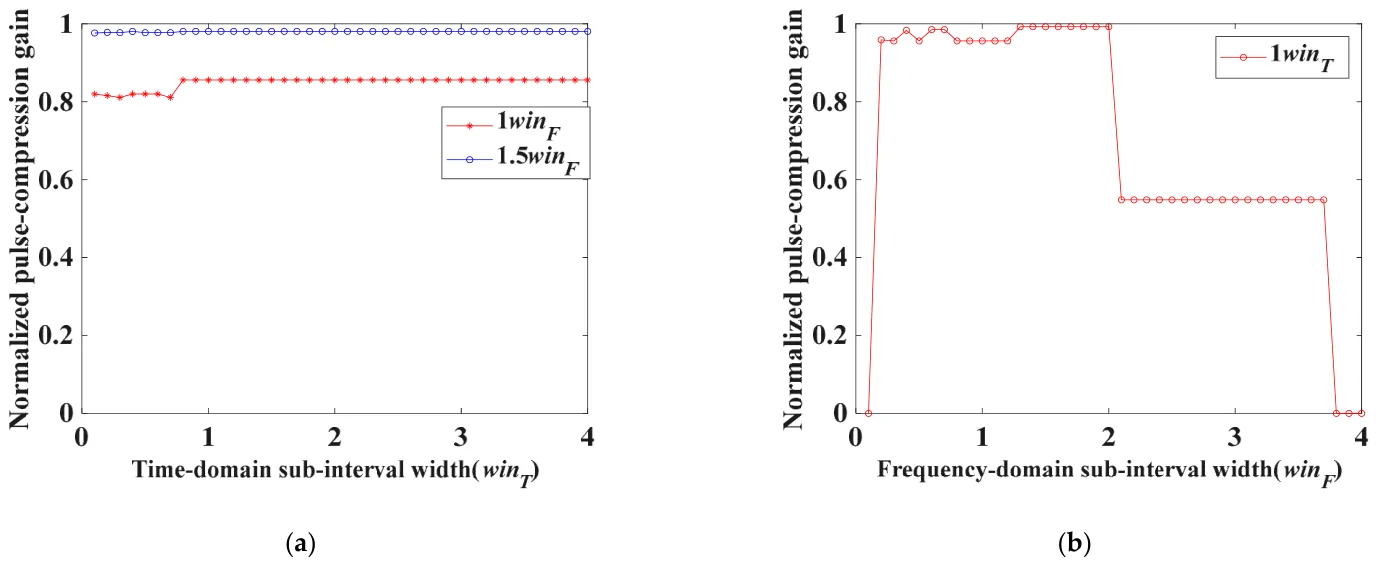
Effect of sub-interval width on pulse-compression gain. (**a**) Pulse-compression gain versus the time-domain sub-interval width. (**b**) Pulse-compression gain versus the frequency-domain sub-interval width.

The method proposed in Section 3 requires designing appropriate filters in both the time and frequency dimensions to remove jamming signals. The selected sub-interval width, particularly in the frequency domain, is critical. The frequency-domain projection is obtained by summing the STFT magnitude over the time axis. Its spread across frequency bins makes threshold selection sensitive to the sub-interval width. This characteristic complicates filter construction. In this section, the method employs a PDF-based procedure to compute the threshold and thereby construct the rejection mask. Its performance is primarily influenced by the selected sub-interval width. Below, the influence of different sub-interval widths on the pulse-compression gain of the filtered signal is analyzed at a JSR of 17.0 dB and an SNR of −7.0 dB. The results are presented in Figure 8.

Figure 8a shows the pulse-compression gain versus the time-domain sub-interval width for frequency-domain widths of 1 *win_F_* and 1.5 *win_F_*. Figure 8b shows the gain versus the frequency-domain sub-interval width for a time-domain width of 1 *win_T_*. The gain is relatively insensitive to the time-domain sub-interval width, whereas the frequency-domain sub-interval width significantly affects the gain, which remains high only over an appropriate range of widths. Appropriate widths in both domains are therefore needed to preserve a high pulse-compression gain.

To provide quantitative guidance for parameter configuration, an offline sensitivity study was conducted in addition to the pulse-compression-gain curves. The dimensionless multipliers *α_T_* and *α_F_* scale the corresponding unit sub-interval widths defined in Section 3.2. Four combinations of SNR and JSR were examined, using the same received data for all parameter choices within each condition. Each multiplier was varied over {0.2, 0.4, 0.6, 0.8, 1.0, 1.25, 1.5, 2.0, 3.0, 4.0, 5.0, 6.0, 8.0} while the other was held fixed. The results of this study are expressed by the peak signal-to-jamming-plus-noise ratio (PSJNR) defined in (28) in Section 4.2.3.

**Figure 9:**
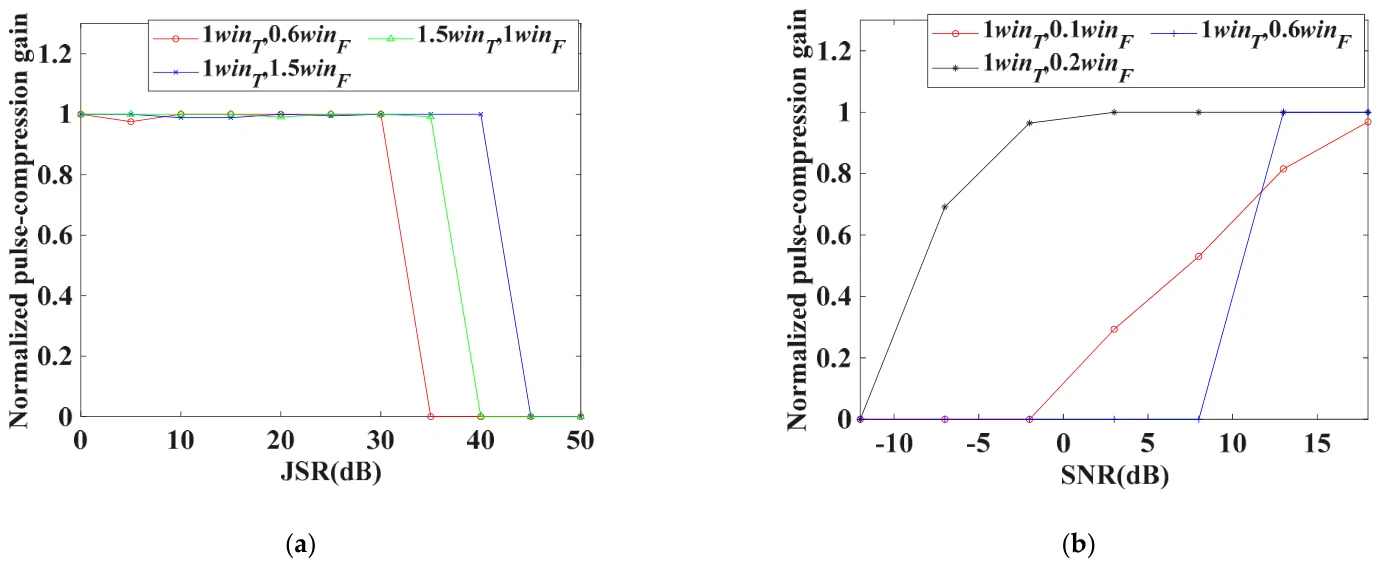
Pulse-compression gain versus (**a**) JSR and (**b**) SNR.

For fixed sub-interval widths, the gain is further affected by the JSR and SNR, as shown in Figure 9. Figure 9a and Figure 9b show the pulse-compression gain versus JSR and SNR, respectively, for different combinations of time- and frequency-domain sub-interval widths. In Figure 9b, the SNR axis extends to an input SNR of 18 dB; at higher SNRs, all tested width combinations retain the full gain.

The pulse-compression gain decreases at high JSRs and low SNRs. In the JSR sweeps that follow, in which the SNR is fixed, the time- and frequency-domain sub-interval widths are set to 1 *win_T_* and 1.5 *win_F_*, respectively. In the SNR sweeps, in which the JSR is fixed, they are set to 1 *win_T_* and 0.2 *win_F_*, respectively.

Table 2 summarizes the PSJNR sensitivity results. With α_F_ fixed at 0.6, varying *α_T_* from 0.2 to 8.0 changed the PSJNR by less than 0.11 dB in each of the four conditions. This weak sensitivity supports α_T_ = 1.0 as a representative setting for analyzing the frequency width.

With α_T_ = 1.0, the PSJNR at SNR = −7 dB and JSR = 17 dB remained approximately 5.13–5.19 dB for the tested α_F_ values between 0.2 and 1.25, but fell to 2.40 dB at α_F_ = 1.5. At SNR = −7 dB and JSR = 27 dB, a comparable plateau extended to α_F_ = 2.0. At SNR = −17 dB and JSR = 27 dB, α_F_ = 0.2 yielded 2.22 dB, whereas α_F_ = 0.4 yielded −2.94 dB. At SNR = −17 dB and JSR = 17 dB, parameter adjustment produced only minor changes and no clear improvement.

These observations support using *α_T_* = 1.0 as a reference and choosing *α_F_* from the performance plateau or best sampled setting observed for the intended operating conditions. A smaller value, such as *α_F_* = 0.2, is a useful candidate when lower-SNR conditions are included. This is empirical guidance from an offline configuration study, rather than an automatic parameter-selection procedure or a guarantee across untested conditions.

In Table 2, the best PSJNR is the maximum over both one-factor sweeps. The frequency-width values refer to the enumerated *α_F_* settings tested with *α_T_* = 1.0. A single best sampled setting is not a plateau, and the ranges do not imply continuous-parameter guarantees.

#### 4.2.2. Jammer Replay Latency Analysis

**Figure 10:**
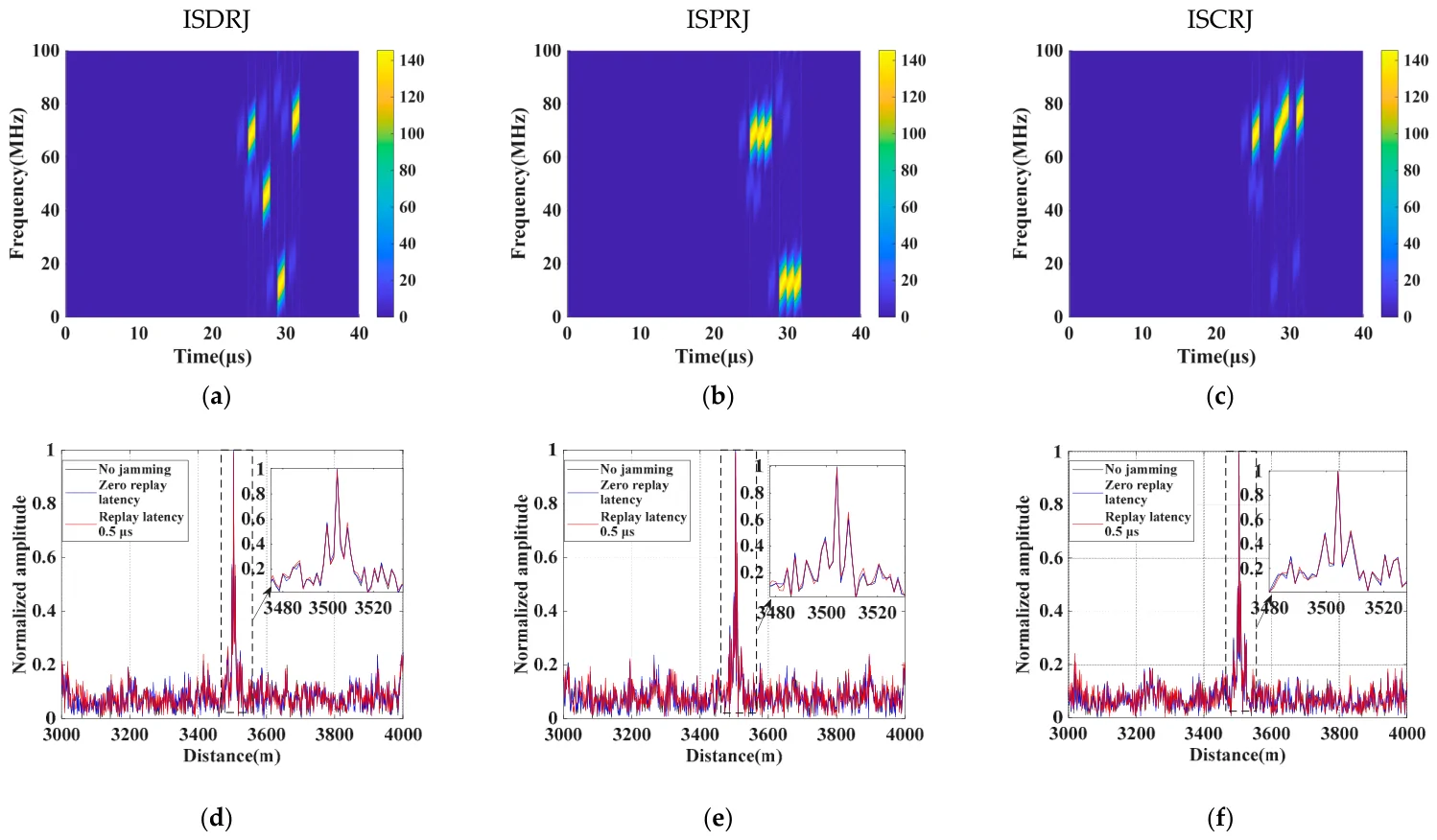
Simulation results with zero and nonzero jammer replay latency. (**a**–**c**) Time–frequency distributions with a replay latency of 0.5 μs: ISDRJ, ISPRJ, and ISCRJ. (**d**–**f**) Pulse-compression results without jamming and after suppression with zero and 0.5 μs replay latency.

**Figure 11:**
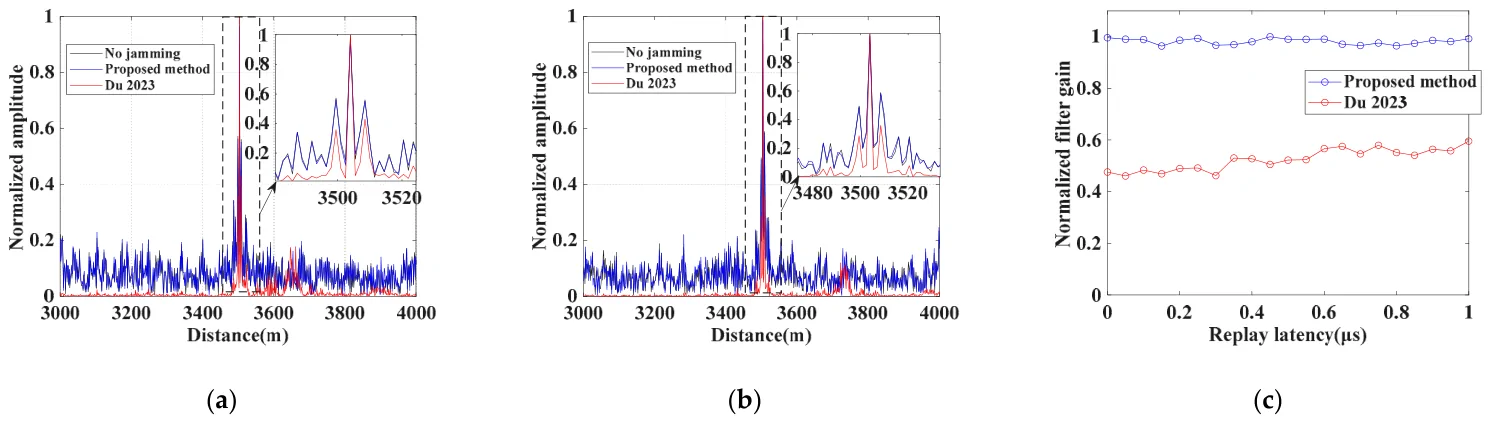
Effect of jammer replay latency on pulse-compression performance. (**a**) Pulse-compression results with zero replay latency. (**b**) Pulse-compression results for a jammer replay latency of *τ*_c_ = 0.5*T*_s_. (**c**) Filter gains versus replay latency.

In the simulations of Section 4.1, the sampling windows of all three ISRJ modes are aligned with the sub-pulses (Table A1). The following analysis varies the additional replay latency of each retransmission while keeping the sampling windows aligned (Table A1). This shifts the already sampled fragments in time; sampling-window offsets that change the contents of a fragment are not simulated. The radar parameters are those in Table 1, and the SNR and the jammer setting are those of Section 4.1. The replay latency is 0.5 μs in Figure 10 and Figure 11b and is varied from 0 to 1 μs in steps of 0.05 μs in Figure 11c. The simulation results are shown in Figure 10. Figure 10a–c show the time–frequency distributions for ISDRJ, ISPRJ, and ISCRJ with a replay latency of *τ*_c_ = 0.5*T*_s_. Figure 10d–f each compare the pulse-compression results for three cases: no jamming, and suppression with zero replay latency and with the specified nonzero replay latency.

Figure 10 shows that the tested replay latency does not cause a marked loss of pulse-compression gain after suppression for any of the three ISRJ modes. In a fixed-filter method, a replay latency shifts the forwarded jamming components and may move their energy outside the predefined rejection region. In the proposed method, the rejection region is estimated from the current time–frequency energy distribution, allowing the mask to track this displacement and maintain suppression performance.

Using ISDRJ as an example, Figure 11 compares the pulse-compression gain obtained with the proposed method and the method in [9] with delayed replay. Figure 11a compares the pulse-compression results of the suppression methods with zero replay latency. Figure 11b presents the corresponding comparison for a jammer replay latency of *τ*_c_ = 0.5*T*_s_. Figure 11c shows the filter gain of each suppression method as a function of replay latency.

The results show that the proposed filters maintain high gain over the tested replay latencies. These tests do not establish robustness to sampling windows that are misaligned with the radar sub-pulses.

#### 4.2.3. JSR Analysis

To quantitatively evaluate jamming suppression, the peak signal-to-jamming-plus-noise ratio (PSJNR) of the post-suppression, pulse-compressed range profile is used. Performance is first assessed as a function of the jamming-to-signal ratio (JSR). The linear power ratio and its decibel form are defined as

(28) $$\delta =\max_{|r-r_{1}|\leq \Delta }{\left|H_{\mathrm{RRP}}(r)\right|}^{2}/\max_{|r-r_{1}|>\Delta }{\left|H_{\mathrm{RRP}}(r)\right|}^{2},\mathrm{PSJNR}=10\log_{10}\delta$$

where *H*_RRP_(*r*) denotes the one-dimensional range profile after suppression and pulse compression, *r*_1_ is the true-target range, and Δ is the half-width of a guard interval set to the main-lobe half-width of the unjammed target response. The numerator is the maximum target-region power, and the denominator is the maximum residual jamming-plus-noise power outside the guard interval. The same Δ is used for all methods and all SNR/JSR settings.

**Figure 12:**
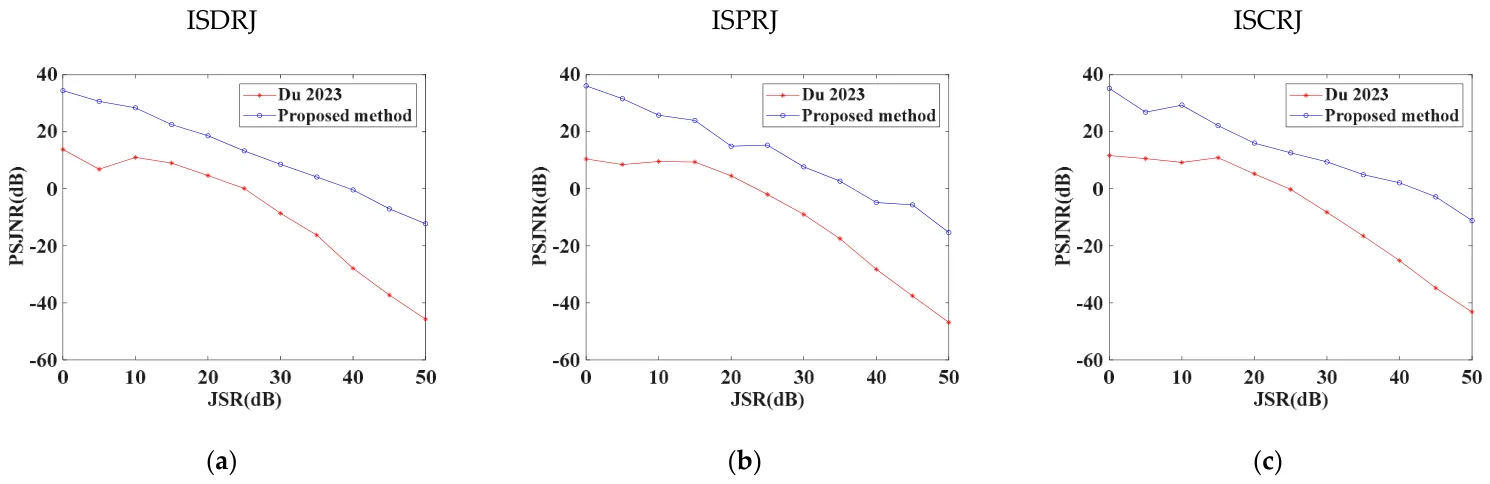
Peak signal-to-jamming-plus-noise ratio (PSJNR) versus jamming-to-signal ratio (JSR). (**a**) ISDRJ. (**b**) ISPRJ. (**c**) ISCRJ.

Using the representative configuration examined in Figure 9, the time- and frequency-domain sub-interval widths are set to 1 *win_T_* and 1.5 *win_F_*, respectively. The PSJNR results as a function of JSR are shown in Figure 12.

Across the three ISRJ modes, the suppression performance of both the proposed method and the method in [9] deteriorates as JSR increases. However, the proposed method maintains a higher PSJNR and remains effective over a wider tested JSR range. It also retains a stronger genuine-target peak, which benefits subsequent target parameter estimation.

#### 4.2.4. SNR Analysis

**Figure 13:**
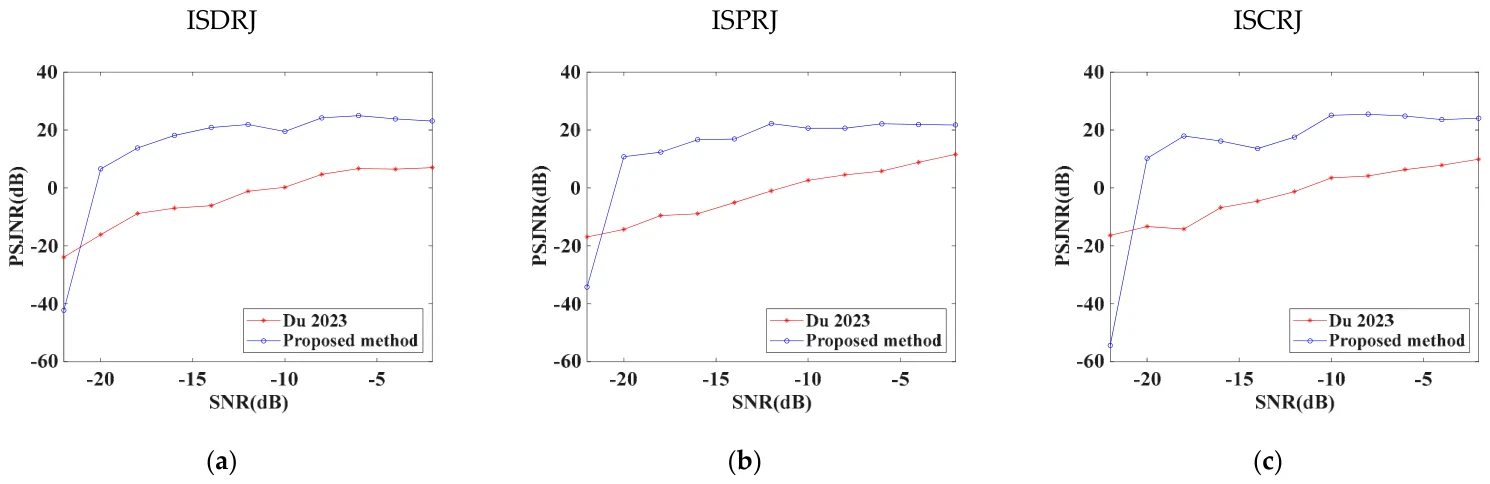
Peak signal-to-jamming-plus-noise ratio (PSJNR) versus signal-to-noise ratio (SNR). (**a**) ISDRJ. (**b**) ISPRJ. (**c**) ISCRJ.

PSJNR is also used to evaluate suppression performance at different SNRs. With the representative configuration examined in Figure 9, the time- and frequency-domain sub-interval widths are set to 1 *win_T_* and 0.2 *win_F_*, respectively. Figure 13 shows the resulting PSJNR as a function of SNR. The tested input SNRs of −22 to −2 dB correspond to 7–27 dB after pulse compression.

### 4.3. Performance Under Non-Ideal Conditions

The preceding simulations use a single jammer, Gaussian receiver noise, and ideal receiver channels. To examine the proposed method further, conditions with range-spread compound-Gaussian clutter, channel gain and phase errors, and a second jammer are added in this subsection.

The clutter is modeled as a range-spread compound-Gaussian return, using Gamma-distributed texture of shape parameter *ν* and complex Gaussian speckle. The resulting reflectivity is convolved with the transmitted sub-pulse and assembled on the same delay grid as the target echo. Texture is held constant over the CPI, while pulse-independent speckle is an explicit simulation assumption. The waveform’s approximately 1.15 GHz inter-pulse frequency span does not by itself establish complete clutter decorrelation. After segmented pulse compression, the simulated clutter has a peak-to-mean ratio of 4.0 dB, compared with 2.4 dB for equal-power white noise, and a lag-one range correlation of 0.21, compared with 0.06; it is therefore waveform-correlated and spread over range. The clutter level is specified by the clutter-to-noise ratio (CNR), defined as the ratio of the mean clutter power to the receiver-noise power at the receiver input over the pulse duration.

**Figure 14:**
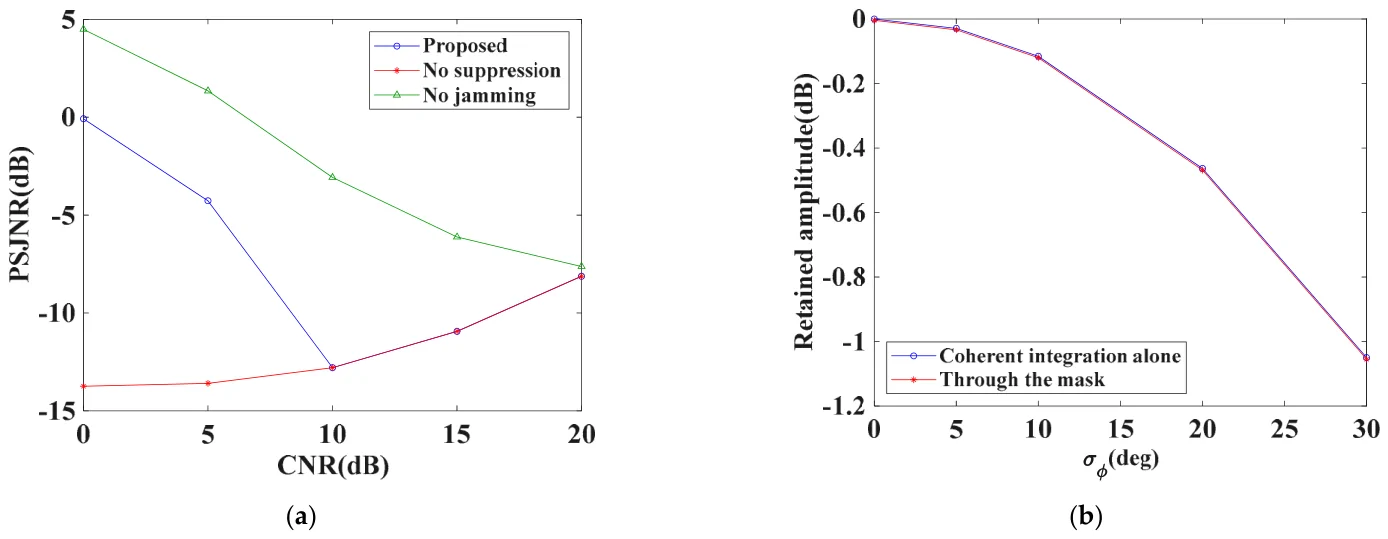
Performance under non-ideal conditions. (**a**) Peak signal-to-jamming-plus-noise ratio (PSJNR) versus the ratio of the mean clutter power to the receiver-noise power (CNR). (**b**) Retained target amplitude versus the standard deviation *σ_φ_* of the channel phase error.

Figure 14a and Table 3 show that clutter reduces the PSJNR improvement over the unfiltered signal from 19.2 dB without clutter to 13.7 and 9.3 dB at CNRs of 0 and 5 dB, respectively. No improvement is observed at CNRs of 10 dB or higher. Target-amplitude loss increases from 0.6 dB without clutter to 5.9 dB at CNR = 5 dB. These results use *α_T_* = 1.0 and, at each CNR, the *α_F_* value among {0.2, 0.4, 0.6, 1.0} that gives the highest PSJNR after suppression.

At texture correlation lengths of 96 and 3072 m, the normalized second moment of clutter intensity is 2.22 and 4.06, respectively, compared with 2.00 for the complex-Gaussian reference. The larger value at 3072 m indicates a stronger departure from Gaussian statistics. The performance limits in Table 3 apply only to the tested compound-Gaussian model (*ν* = 4).

For the channel-error tests in Figure 14b and Table 4, independent gain or phase errors are applied to each sub-pulse of the target-plus-jamming signal before adding receiver noise. Filtering adds 0.01 dB of target-amplitude loss relative to coherent integration alone at a phase-error standard deviation *σ_φ_* of 30° or a gain-error standard deviation of 30% of nominal amplitude. Small amplitude losses do not imply an unchanged filtering mask. All amplitudes are normalized to the unfiltered, error-free, target-only reference; positive values under gain errors reflect amplitude increases in the tested realization.

In the two-jammer test, a second return is formed by shifting a copy of the first jammer return by 450 m in equivalent range (an additional 3 μs delay) and applying a phase that is independently and uniformly drawn over [0, 2π) for each pulse. The two returns use the same ISDRJ forwarding schedule and per-jammer JSR; the sub-interval widths are selected using the same search as in Table 3. The reported PSJNR improvement is 19.2 dB, without requiring the jammer count as input. This test and the replay-latency tests in Section 4.2.2 support performance only under the specified configurations; robustness to general model mismatch remains unverified.

### 4.4. Comparison with Other Suppression Methods

All numerical comparisons use identical simulated received signals, waveform parameters, and SNR/JSR settings. For each trial, PSJNR is calculated from (28) with a guard interval of one range-resolution cell and summarized by its median over the CPI. The reported values are medians across three independent trials.

Reference [9] applies an Otsu threshold to the time-direction projection of the STFT of the complete received signal. The proposed method uses both time and frequency projections with a threshold derived from the modal histogram interval. To separate these effects, Otsu, Kittler–Illingworth, and two-means thresholds are also evaluated in the same two-dimensional framework, with only the threshold rule changed. Two-means serves as a threshold baseline rather than a complete ISRJ recognition system.

**Figure 15:**
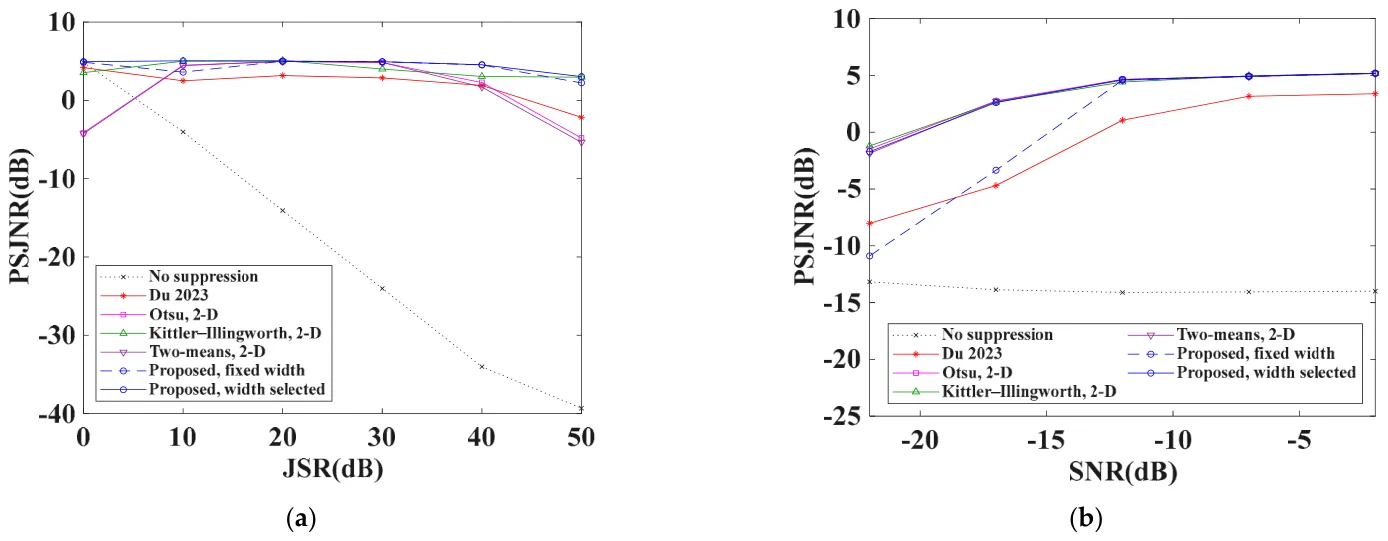
PSJNR comparison versus (**a**) JSR and (**b**) SNR.

In Figure 15, the two-dimensional variants generally outperform [9] and differ by about 1 dB or less over most of the tested range. This supports the benefit of joint time and frequency filtering, while threshold differences become more pronounced near the limits of the JSR range.

Otsu and two-means maximize between-class variance and minimize within-cluster variance, respectively, without testing for a distinct jamming population. At JSR = 0 dB, they remove some target components and yield PSJNRs of −4.13 and −4.25 dB, respectively, versus 4.88 dB without filtering and 4.94 dB for the proposed rule (widths selected offline; 4.88 dB with the fixed widths *α_T_* = 1.0 and *α_F_* = 0.6). At JSR = 50 dB, strong jamming raises their thresholds and leaves more residual jamming; PSJNR falls to −4.80 and −5.34 dB, versus 3.03 dB for the proposed rule (2.24 dB with fixed widths). All threshold variants still improve on the unfiltered value of −39.30 dB. Table 5 summarizes the criteria, including the two-Gaussian assumption of Kittler–Illingworth. The proposed rule avoids fitting a second class but requires the modal interval to represent the dominant low-amplitude background, which limits the applicability of these results.

The proposed rule requires a sub-interval width. At an input SNR of −22 dB, its fixed-width PSJNR is −10.89 dB, compared with −1.48 dB for Otsu. Selecting the best of eight tested width-multiplier pairs offline at each operating point raises this value to −1.71 dB and keeps the selected-width curve within 0.5 dB of the best parameter-free rule across the tested SNR range. Its benefit is therefore robustness across JSR with suitable tuning, rather than uniformly higher PSJNR.

For a 64-pulse CPI on the same machine, runtimes are 1.5 s for [9], 9.1 s for the proposed method, and 9.0, 9.0, and 9.3 s for the two-dimensional Otsu, Kittler–Illingworth, and two-means variants, respectively. The two-dimensional variants share the STFT and inverse STFT for each sub-pulse and show little runtime variation with threshold choice. These unoptimized single-pass timings use preset widths, exclude offline search, and do not establish real-time capability.

Compared with sparse reconstruction [31], the proposed method constructs rejection masks directly from time–frequency projections without estimating jammer sampling or forwarding parameters. It therefore avoids dictionary construction, iterative recovery, and the propagation of reconstruction errors into cancellation, allowing the mask to follow replay-induced displacements when target and jamming remain sufficiently separable (Section 4.2.2). Compared with the learning-based method in [32], the proposed method estimates its thresholds directly from the received signal and requires no training data or pretrained weights. This makes it suitable for frequency-agile radar when representative training data are unavailable.

These alternatives also carry practical limitations for the scenario considered here. Sparse-reconstruction methods such as [31] estimate the ISRJ parameters over a discretized dictionary and then reconstruct and cancel the jamming. When the jammer samples asynchronously, its sampling instants need not coincide with the dictionary grid; the resulting dictionary mismatch can degrade the parameter estimates, and the reconstruction error then propagates into the cancellation. Learning-based methods such as [32] depend on sufficient training data matched to the waveform, jamming mode, SNR, and JSR encountered in operation, and their performance outside the training conditions is not assured. Quantitative benchmarking under identical simulation conditions will be completed in future work.

The runtimes above can be related to the computational complexity. Let *L* denote the number of samples in one pulse repetition interval, *K* the number of STFT frames, and *N_F_* the length of the fast Fourier transform (FFT). The proposed method computes one STFT and one inverse STFT for each of the *N_S_* sub-pulse channels of each of the *M* pulses, which requires *O*(*MN_S_KN_F_*log*N_F_*) operations; forming the projections and applying the masks requires *O*(*MN_S_KN_F_*), the histogram thresholds require *O*(*MN_S_*(*K* + *N_F_*)), and segmented pulse compression requires *O*(*MN_S_L*log*L*). The method in [9] computes one STFT and one inverse STFT per pulse, *O*(*MKN_F_*log*N_F_*), followed by the same segmented pulse compression.

The proposed method is therefore more expensive by a factor of about *N_S_* in the transform stage, which is the price of the sub-pulse-wise two-dimensional mask. With *N_S_* = 8, the measured runtime ratio of 9.1 s to 1.5 s is consistent with this estimate, because the pulse-compression stage is common to both methods. Replacing the threshold criterion changes only the *O*(*MN_S_*(*K* + *N_F_*)) term, which explains the nearly equal runtimes of the two-dimensional variants. Sparse-reconstruction methods add an iterative recovery whose cost grows with the dictionary size and the number of iterations, and learning-based methods move most of the cost to offline training; their runtimes were not measured here. Table 6 summarizes the comparison.

## 5. Discussion

The results indicate that the proposed method suppresses ISRJ primarily by exploiting the joint time–frequency displacement between forwarded jamming components and genuine target echoes. Compared with single-domain filtering, the proposed method applies both time- and frequency-domain criteria to each sub-pulse to localize jamming-contaminated cells while retaining target-bearing components. The empirical probability-density criteria reduce reliance on a fixed global threshold, contributing to stable performance as SNR, JSR, and jammer replay latency vary. The results for ISDRJ, ISPRJ, and ISCRJ suggest that the method exploits a characteristic shared by the three forwarding schedules rather than one specific mode. For target detection with frequency-agile radar remote-sensing systems, preserving target-bearing components before segmented pulse compression helps retain the information needed for subsequent detection and estimation of range and velocity.

Section 4.3 shows a PSJNR benefit in the tested compound-Gaussian clutter at CNRs up to 5 dB, with about 5.9 dB of target-amplitude loss at 5 dB. Under the tested conditions, the proposed method no longer improves PSJNR relative to the unfiltered signal when the CNR reaches 10 dB or higher. Channel-error tests show an incremental target-amplitude loss of 0.01 dB at a phase-error standard deviation of 30° or a gain-error standard deviation of 30%. These small observed losses do not establish general mask invariance. Non-Gaussian receiver noise and general model mismatch remain unvalidated; the replay-latency and two-jammer tests cover only the stated configurations. Sampling-window offsets relative to the radar sub-pulses remain unvalidated. The evidence remains simulation-based and uses joint intra-pulse and inter-pulse frequency agility. The waveform parameters were fixed to those in Table 1. The method relies on the frequency separation between a forwarded fragment and the overlapped sub-pulse being resolvable by the STFT; a smaller frequency step or a shorter sub-pulse relative to the STFT resolution is expected to reduce this separability, and the dependence on the waveform parameters remains to be quantified.

The simulations in this paper assume complex Gaussian receiver noise, whereas receiver noise can also be non-Gaussian, for example, impulsive or heavy-tailed. Isolated high-amplitude noise samples can then distort the time and frequency projections and the histogram-based thresholds derived from them, so that residual jamming may remain or target-bearing cells may be rejected; the evaluation of this effect is identified as a follow-up research item.

Future work will examine cascading the method with clutter cancellation, evaluating its performance under non-Gaussian receiver noise, repeating the evaluation for other waveform parameter sets, extending the offline parameter-sensitivity study to multiple jammers of comparable power, and validating these configurations using measured frequency-agile radar data. Quantitative benchmarking against representative sparse-reconstruction and learning-based methods will also be completed under identical simulation conditions; for the learning-based methods, it will use matched waveform conditions and independently generated training, validation, and test realizations.

## 6. Conclusions

This paper establishes a unified received-signal model for a frequency-agile radar under three ISRJ forwarding modes and develops a suppression method based on time–frequency analysis and multi-domain filtering. Empirical probability-density criteria derived from the time and frequency projections of each sub-pulse are used to construct two-dimensional rejection masks. The retained target-bearing components are reconstructed by the inverse STFT and processed using segmented pulse compression. Simulations with aligned sampling windows and zero or nonzero replay latency show that the method suppresses coherent false targets while limiting pulse-compression-gain loss and achieving higher PSJNR than the reference method across the tested SNR and JSR ranges. The method therefore has potential as an interference-mitigation step before target detection in frequency-agile radar remote-sensing systems operating in contested electromagnetic environments.

## Figures and Tables

**Table 1:** Simulation parameters.

Parameter	Value	Parameter	Value
Initial carrier frequency/GHz	9	Sub-pulse width/μs	1
Sub-pulse bandwidth/MHz	6	Pulse repetition frequency/kHz	25
Sampling frequency/MHz	100	Number of pulses	64
Number of sub-pulses	8	Sub-pulse frequency step/MHz	5
Frequency-agility codebook size	128	Inter-pulse frequency step/MHz	9
Target distance/m	3503.8	Target speed/(m/s)	41.8

**Table 2:** Offline PSJNR sensitivity to the sub-interval-width multipliers at four operating points.

Operating Point	PSJNR Without Filtering/dB	Best PSJNR/dB	Tested *α_F_* Plateau/Best Sampled Value
SNR −7.0 dB, JSR 17.0 dB	−13.96	5.19	0.2–1.25
SNR −7.0 dB, JSR 27.0 dB	−23.99	5.22	0.2–2.0
SNR −17.0 dB, JSR 27.0 dB	−13.85	2.22	0.2 (best sampled value)
SNR −17.0 dB, JSR 17.0 dB	−4.08	−3.90	No clear improvement

**Table 3:** Performance in range-spread compound-Gaussian clutter.

CNR/dB	PSJNR, No Filtering/dB	PSJNR, After Suppression/dB	PSJNR, No Jamming/dB	Target Amplitude Retained/dB
no clutter	−13.96	5.19	5.15	−0.64
0	−13.74	−0.08	4.50	−5.79
5	−13.59	−4.27	1.34	−5.93
10	−12.79	−12.79	−3.07	0.00
15	−10.93	−10.93	−6.12	0.00
20	−8.12	−8.12	−7.62	0.00

CNR: clutter-to-noise ratio; PSJNR: peak signal-to-jamming-plus-noise ratio.

**Table 4:** Effect of channel gain and phase imbalance on the target amplitude.

Error Standard Deviation	Coherent Integration Alone/dB	Through the Suppression Mask/dB	Incremental Loss/dB
Phase, 10°	−0.12	−0.12	0.00
Phase, 20°	−0.46	−0.47	0.01
Phase, 30°	−1.05	−1.06	0.01
Gain, 10%	0.07	0.07	0.00
Gain, 20%	0.14	0.13	0.01
Gain, 30%	0.21	0.20	0.01

The error standard deviation is given in degrees for the phase error (*σ_φ_* in Figure 14b) and as a percentage of the nominal amplitude for the gain error.

**Table 5:** Threshold criteria and PSJNR at JSR = 0 and 50 dB.

Strategy	Criterion	Free Parameter	JSR 0 dB	Strong Jamming, JSR 50 dB
Modal interval, proposed	upper boundary of the sub-interval of largest empirical density	sub-interval width	4.94 dB; unfiltered: 4.88 dB	3.03 dB
Otsu, 2-D baseline	maximum between-class variance	none	−4.13 dB, target partly discarded	−4.80 dB, threshold raised by the jamming
Kittler–Illingworth	minimum classification error, two-Gaussian model	none	3.54 dB	2.97 dB
Two-means clustering	minimum within-cluster variance	none	−4.25 dB, target partly discarded	−5.34 dB, threshold raised by the jamming

PSJNR: peak signal-to-jamming-plus-noise ratio; JSR: jamming-to-signal ratio; 2-D: two-dimensional. The Otsu row refers to the two-dimensional variant evaluated here; the method in [9] uses the same criterion in a one-dimensional filter. Jamming is present at JSR = 0 dB.

**Table 6:** Computational complexity and measured runtime of the compared methods.

Method	Transform Stage	Threshold Stage	Runtime per CPI/s
Reference [9]	*O*(*MKN_F_*log*N_F_*)	*O*(*MK*)	1.5
Proposed	*O*(*MN_S_KN_F_*log*N_F_*)	*O*(*MN_S_*(*K* + *N_F_*))	9.1
Otsu, 2-D	*O*(*MN_S_KN_F_*log*N_F_*)	*O*(*MN_S_*(*K* + *N_F_*))	9.0
Kittler–Illingworth, 2-D	*O*(*MN_S_KN_F_*log*N_F_*)	*O*(*MN_S_*(*K* + *N_F_*))	9.0
Two-means, 2-D	*O*(*MN_S_KN_F_*log*N_F_*)	*O*(*MN_S_*(*K* + *N_F_*))	9.3
Sparse reconstruction [31]	Iterative recovery; cost grows with the dictionary size and the number of iterations	–	Not measured
Learning-based [32]	Network inference; offline training required	–	Not measured

*M*: number of pulses in a CPI (64); *N_S_*: number of sub-pulses (8); *L*: samples per pulse repetition interval (4000); *K*: STFT frames per pulse (499); *N_F_*: FFT length (2048). All methods in the first five rows additionally require segmented pulse compression, *O*(*MN_S_L*log*L*). Runtimes are single-pass values on the same machine with preset widths and exclude the offline width search. 2-D: two-dimensional.

## Data Availability

The data are available upon request due to restrictions.

## Abbreviations

The following abbreviations are used in this manuscript:

CNR	Clutter-to-noise ratio
CPI	Coherent processing interval
DRFM	Digital radio frequency memory
FFT	Fast Fourier transform
ISCRJ	Interrupted-sampling cyclic repeater jamming
ISDRJ	Interrupted-sampling direct repeater jamming
ISPRJ	Interrupted-sampling periodic repeater jamming
ISRJ	Interrupted-sampling repeater jamming
ISTFT	Inverse short-time Fourier transform
JSR	Jamming-to-signal ratio
LFM	Linear frequency modulation
PDF	Probability density function
PSJNR	Peak signal-to-jamming-plus-noise ratio
SNR	Signal-to-noise ratio
STFT	Short-time Fourier transform

## Appendix A

Table A1 lists the sampling and forwarding settings used in Section 4. The sampling windows are aligned with the sub-pulses, so that each sampled fragment is one whole sub-pulse; the sub-pulses and the sub-pulse intervals of the received pulse are numbered from 1 to 8. Each fragment is retransmitted in a later interval of the same pulse, starting a replay latency after the beginning of that interval, so that its forwarding delay is the separation between the sampled sub-pulse and the interval plus the replay latency. A replay for which no interval is left in the pulse is not transmitted. Section 4.2.2 varies the replay latency while keeping the sampling windows fixed and aligned with the sub-pulses. In the single-jammer tests, the fragments carry the target’s round-trip propagation delay and a common amplitude set by the JSR, with the same schedule in every pulse. The two-jammer test in Section 4.3 adds a second return with an extra 3 μs propagation delay (450 m in equivalent range), the same forwarding schedule and per-jammer JSR, and an independent uniformly distributed phase in each pulse. The sampling durations and periods of ISDRJ and ISPRJ, and the forwarding count of ISPRJ, follow the reference method [9].

**Table A1:** ISRJ settings used in the simulations.

Parameter	ISDRJ	ISPRJ	ISCRJ
Sampling duration *T*_sam_/μs	1	1	1
Sampling period *T_J_*/μs	2	4	3
Duty factor *T*_sam_/*T_J_*	1/2	1/4	1/3
Sampled sub-pulses	1, 3, 5, 7	1, 5	1, 4, 7
Retransmissions (sub-pulse → interval)	1 → 2, 3 → 4, 5 → 6, 7 → 8	1 → 2, 3, 4; 5 → 6, 7, 8	1 → 2; 1 → 5, 4 → 6; 4 → 8
Forwarding count	1	*Q* = 3	*C* = 2
Forwarding delays at zero latency/μs	1	1, 2, 3	1; 4, 2; 4
Replay latency/μs	0	0	0 (0.5 in Section 4.1)
